# Deciphering the functional specialization of whole-brain spatiomolecular gradients in the adult brain

**DOI:** 10.1073/pnas.2219137121

**Published:** 2024-06-11

**Authors:** Jacob W. Vogel, Aaron F. Alexander-Bloch, Konrad Wagstyl, Maxwell A. Bertolero, Ross D. Markello, Adam Pines, Valerie J. Sydnor, Alex Diaz-Papkovich, Justine Y. Hansen, Alan C. Evans, Boris Bernhardt, Bratislav Misic, Theodore D. Satterthwaite, Jakob Seidlitz

**Affiliations:** ^a^Department of Clinical Sciences Malmö, SciLifeLab, Lund University, Lund, Sweden 202 13; ^b^Lifespan Informatics and Neuroimaging Center, University of Pennsylvania, Philadelphia, PA 19104; ^c^Department of Psychiatry, University of Pennsylvania, Philadelphia, PA 19104; ^d^Department of Child and Adolescent Psychiatry and Behavioral Science, The Children’s Hospital of Philadelphia, Philadelphia, PA 19104; ^e^Penn-Children’s Hospital of Philadelphia Lifespan Brain Institute, University of Pennsylvania, Philadelphia, PA 19104; ^f^Wellcome Centre for Human Neuroimaging, Institute of Neurology, University College London, London WC1N 3AR, United Kingdom; ^g^McConnell Brain Imaging Centre, Montreal Neurological Institute, McGill University, Montreal, QC H3A 2B4, Canada; ^h^Department of Psychiatry and Behavioral Sciences, Stanford University, Stanford, CA 94305; ^i^Quantitative Life Sciences, McGill University, Montreal, QC H3A 1E3, Canada; ^j^McGill Genome Centre, McGill University, Montreal, QC H3A 0G1, Canada

**Keywords:** transcriptomics, arealization, neuroimaging, morphogen

## Abstract

Cortical arealization arises during neurodevelopment from the confluence of molecular gradients representing patterned expression of morphogens and transcription factors. However, whether similar gradients are maintained in the adult brain remains unknown. Here, we uncover three axes of topographic variation in gene expression in the adult human brain that specifically capture previously identified rostral-caudal, dorsal-ventral, and medial-lateral axes of early developmental patterning. The interaction of these spatiomolecular gradients i) accurately reconstructs the position of brain tissue samples, ii) delineates known functional territories, and iii) can model the topographical variation of diverse cortical features. The spatiomolecular gradients are distinct from canonical cortical axes differentiating the primary sensory cortex from the association cortex, but radiate in parallel with the axes traversed by local field potentials along the cortex. We replicate all three molecular gradients in three independent human datasets as well as two nonhuman primate datasets and find that each gradient shows a distinct developmental trajectory across the lifespan. The gradients are composed of several well-known transcription factors (e.g., *PAX6* and *SIX3*), and a small set of genes shared across gradients are strongly enriched for multiple diseases. Together, these results provide insight into the developmental sculpting of functionally distinct brain regions, governed by three robust transcriptomic axes embedded within brain parenchyma.

## Significance

During brain development, morphogens diffuse through the brain in gradient-like patterns, helping to define distinct regions with specific functional roles. We identify and replicate three different patterns of gene expression in the adult brain that resemble these developmental gradients. These same expression patterns are present in other mammalian species, and their intersection outlines the main functional divisions of the cerebral cortex. Furthermore, we show that these gene expression gradients are observable throughout neurodevelopment, even if the genes that compose them change. Together, we show gradients to be a central principle underlying spatial gene expression, and we build associative evidence for their functional relevance in adults.

The brain coordinates different facets of behavior through diverse cell populations that are spatially distinct yet anatomically connected through a network of white matter fibers. This topographical distribution of function in the brain emerges during early development through the process of arealization, where cell differentiation and migration create a mosaic of brain areas or subdivisions with distinct molecular properties (1, 2). One of the key tenets of arealization is the expression of morphogen gradients along the cardinal axes of the developing brain. Morphogens are produced from patterning centers and diffuse along developing neural compartments, forming semiorthogonal concentration gradients as the forebrain emerges (3–5). Morphogen expression triggers a cascade of molecular differentiation leading to the formation of spatially distributed gradients (6). These large-scale molecular gradients establish an organizational scaffold where areas distant from one another are also distinct in their molecular (and emergent functional) properties (2).

Molecular gradients have been previously described in the developing brain, namely those that radiate along rostral-caudal (longitudinal), dorsal-ventral (vertical), or medial-lateral (horizontal) axes of the neural compartment (7, 8). Confluent spatial expression of these gradients in turn defines important divisions within the brain along those same axes, which appear to be strongly conserved across phylogeny (9). Cerebral organoids, which model early prenatal development (10), will spontaneously self-organize along similar spatial axes (11). Furthermore, the establishment of molecular patterning gradients appears to be a critical component of normal brain development, as variations to genes regulating these gradients can cause developmental malformations, many of which cause severe morbidity or mortality (12). While many of these findings have been discovered using nonhuman model systems, they have largely been validated by studies using postmortem human tissue (13, 14) or induced pluripotent stem cells (15, 16).

Recent work has also focused on how molecular gradients may help to define the functional organization of the cerebral cortex, which exhibits substantially less molecular differentiation and a more uniform physiology compared to other parts of the brain (17). Nonetheless, the cerebral cortex exhibits functionally distinct territories with subtle variations in physiology (18) and can be further subdivided into smaller regions with ostensibly differentiated roles in cognition and behavior (19). A robust functional hierarchy has been described that situates different cortical brain regions along a sensorimotor–association axis (20–24). Besides discriminating functional roles of different cortical territories, variation in other physiological properties of the cortex occurs along a similar topography to the functional hierarchy (23, 25–27), and it may serve as a principal avenue for directional functional signals (28–31). Cortical peaks in the functional hierarchy are distributed nearly maximally distant from one another (20, 32), suggesting that the functional hierarchy may arise due to interacting underlying transcriptomic gradients (33). Furthermore, several studies have used transcriptomic data densely sampled across the adult cortex to show principal components of cortical gene expression and have noted their topographic similarity to the functional hierarchy (23, 34, 35). What is not entirely clear is whether and how the morphogenetic gradients that give rise to early developmental patterning relate to the adult functional hierarchy, nor the degree to which these relationships are present in other mammals.

Although previous studies have identified heterochronicity in the spatiotemporal expression of many genes in the human brain (36, 37), prior work has not investigated whether developmental directional gradients of gene expression are still present in adulthood, and whether or how they relate to functional specialization. Regional development of the cerebral cortex appears to occur along specific directional gradients in terms of peak growth and rate of change (38). Furthermore, heritability analyses suggest a genetic basis to the organization of cortical surface area and thickness along principal anterior-posterior and dorsal-ventral aspects, respectively (39–41). Follow-up work has suggested that these genetically imposed patterns are disrupted in developmental disorders (42, 43). More recent complementary work has provided evidence for an isolated directional gradient of gene expression in the adult brain, relating this gradient to other brain (neurodegenerative) diseases (44). Together, these studies suggest directional developmental molecular gradients may be present in the adult brain, are distinct from the functional hierarchy, and may be relevant for brain-related traits and disorders. However, whether and how transcriptional gradients in the adult brain relate to molecular gradients underlying early fetal brain development remains unclear.

In the present study, we used a multivariate data-driven approach to establish directional gradients of gene expression in the adult human brain that vary along the axes of morphogen gradients in the developing brain. Unlike most previous studies, we sample directional gene expression across the entire brain, not just the cerebral cortex, to match the propagation of gradients through the developing fetal brain. We validate the presence of identified gradients in three independent datasets of postmortem adult human tissue, track their evolution over the course of development, and examine their phylogenetic stability by examining the presence of these gradients in the brains of nonhuman primates and mice. Importantly, we show that whole-brain gradients converge in the cerebral cortex to form recognizable divisions representing established cytoarchitectonically defined cortical territories. Finally, we show that genes involved in the coordination of multiple gradients are specifically associated with brain diseases, underscoring the importance of molecular gradients as organizational foundations for the healthy brain. Together, our results are suggestive of conserved developmental gradients that are transcriptomically encoded in the adult brain, and which may represent an important component of adult cortical differentiation.

## Results

### Spatial Transcriptomic Gradients in the Adult Human Brain.

**Fig. 1.**
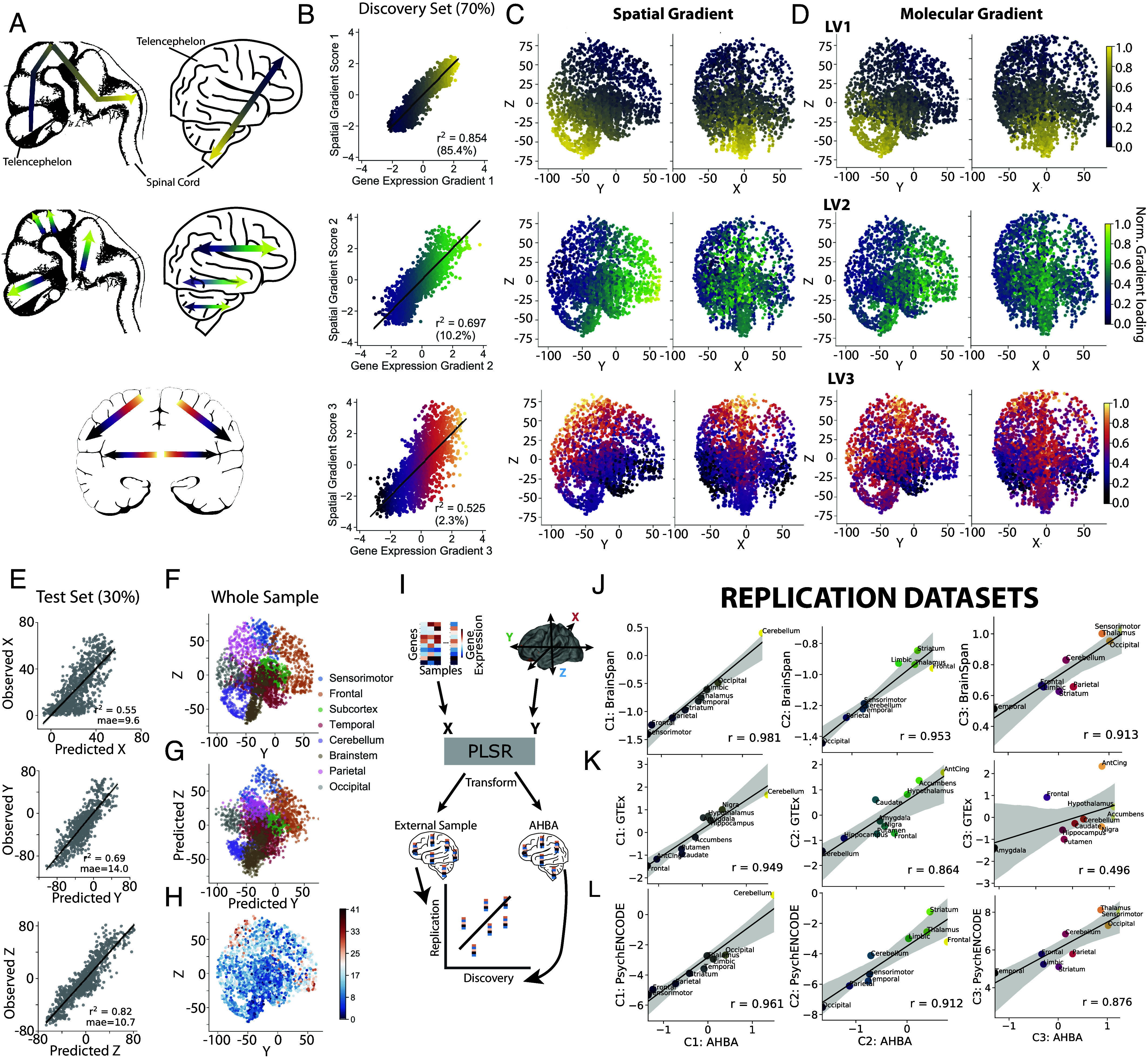
Large-scale spatiomolecular axes of the adult human brain. Partial least squares (PLS) analysis finds strong covariance between gene expression and three-dimensional spatial coordinates, summarized by three components resembling previously described whole-brain developmental molecular gradients. (*A*) Theoretical whole-brain molecular gradients of the developing human brain, adapted (with permission) from Flores-Sarnat and Sarnat (12), and corresponding theoretical projections of these same gradients onto the adult brain. This includes (*Top*) a rostral-caudal gradient extending between the forebrain and hindbrain, (*Middle*) a dorsal-ventral gradient traversing each compartment, and (*Bottom*) dorsomedial-ventrolateral (horizontal) gradient in the telencephalon. (*B*) A PLS regression model was used to link whole-brain gene expression with Euclidean spatial coordinates in a training sample representing 70% of the total dataset (*Materials and Methods* and *SI Appendix*, Fig. S1*A*). This model derived gradients of systematic linear variation in gene expression across spatial axes. Scatter plots show each of the three PLS LVs in the form of correlations between latent spatial and transcriptomic variables. (*C*) Representation of the latent spatial variables of, from left-to-right, LV1, LV2, and LV3. These latent variables represent the main linear axes along which gene expression covaries through space. (*D*) Representation of the latent transcriptomic variables of LV1, LV2, and LV3. These latent variates show the matching patterns of gene expression that vary along axes defined in (*C*). Note that correlations between (*C*) and (*D*) are represented by the scatter plots in (*B*). (*E*) The three latent PLS variables were together able to predict the spatial coordinates (from top-bottom, x, y, and z) of unseen tissue samples based on each sample’s gene expression data alone. The unseen samples consisted of 30% of tissue samples left out of the PLS model fitting. (*F*) Y and Z coordinates of all Allen Brain Atlas tissue samples. (*G*) PLS-predicted Y and Z coordinates of the same samples. (*H*) Plot showing distribution of prediction error in space (average of 11.4 mm). (*I*) The PLS model was refitted using only genes shared across discovery and replication datasets to allow us to test the reliability of the three spatiomolecular axes in entirely new data. This model was used to transform gene expression from the discovery and replication tissue samples into latent transcriptomic variables that compose the PLS LVs. Regional expression of each component in the discovery dataset was correlated against regional expression in the replication dataset. The observed regional correlation between AHBA PLS LVs and external PLS LVs indicates the degree to which regional expression of each component is similar across datasets. A high correlation indicates a similar gradient across a similar spatiomolecular axis exists in the external data. Dataset correlation is averaged over adults from the (*J*) Brainspan (n = 6), (*K*) GTEx (n = 121), and (*L*) PsychENCODE (n = 6) datasets. The observed spatiomolecular axes were highly consistent across datasets with the exception of LV3 expression in GTEx.

We hypothesized that large-scale developmental transcriptomic gradients (Fig. 1*A*) would be detectable in the adult human brain as directional autocorrelation of gene expression spanning the entire brain. To delineate adult transcriptomic gradients, we cross-decomposed a correlated expression matrix composed of 15,634 genes across 3,466 tissue samples (*SI Appendix*, *Methods*) with a matrix of three-dimensional Euclidean coordinates for the same tissue samples (*SI Appendix*, Fig. S1*A*). The resulting components represent the principal sources of directional linear variation in whole-brain gene expression. In other words, the gradients represent sets of key genes that become more and more different as one moves in one direction across the brain. A three-component partial least squares (PLS) regression (45) model provided the best reconstruction of sample location based on repeated 10-fold cross-validation on training data (70% of samples) (*SI Appendix*, Fig. S1*C* and Fig. 1*B*). Tissue expression across the three transcriptomic PLS latent variables (LV1, LV2, LV3) explained the majority of variance across all three Euclidean coordinates of left-out tissue samples (X: R^2^ = 0.55, Y: R^2^ = 0.69, Z: R^2^ = 0.82) and were sufficient to predict the location of left out tissue samples (test data; 30% of samples) extracted throughout the brain with an average of 11.4 mm error (Fig. 1 *E* and *H*). The compositions of the gene expression LVs were also largely invariant to spatial permutations of the brain (*SI Appendix*, Fig. S1*F*).

Having demonstrated robust generalizability, the PLS model was then fitted using all data and was used to reconstruct the location of all tissue samples (Fig. 1 *F* and *G*; see Dataset S1 for component-weighted genes). Based on these findings, the PLS latent variables can collectively be considered as a set of three-dimensional spatiomolecular axes along which brain regions are putatively organized or embedded. Notably, reconstruction was least accurate for tissue samples extracted from the polar extremes of frontal, parietal, and temporal cortex (Fig. 1*H* and *SI Appendix*, Fig. S3). While this phenomenon may be a byproduct of linear modeling, it may also relate to the relatively limited variance of gene expression across cortical areas (17, 37) or to the extreme expansion of these regions among primates (46); these three scenarios are not mutually exclusive.

Despite being generated using a data-driven approach, the three PLS latent variables closely resembled theorized developmental whole-brain spatial gene expression gradients (Fig. 1 *A*, *C*, and *D*). These patterns could not be explained by regional variation in aggregate gene expression across samples (*SI Appendix*, Fig. S4*B*). See *SI Appendix*, *Results* and Fig. S4 for further characterization of each gradient.

### Transcriptomic Gradient Expression Is Consistent across Adult Human Datasets.

While spatially comprehensive, the discovery dataset for the three transcriptomic gradients was composed of gene expression data from only six individuals. Therefore, we sought to test the degree to which expression of these gradients generalized to other datasets of brain gene expression. We used the PLS model trained in the discovery dataset (AHBA) to transform the expression of the molecular gradients in the other replication datasets, GTEx, BrainSpan and PsychENCODE (Fig. 1*I*). We verified the efficacy of these predictions by validating that the gradients had similar expression profiles across spatially distributed brain regions. We found excellent reproducibility of all three gradients when averaging across adult individuals, despite different brain regions being measured across datasets (Fig. 1 *J*–*L*). In general, replication of both LV1 (range: r = 0.95 to 0.98) and LV2 (r = 0.86 to 0.95) were quite strong, while LV3 replication was more variable (r = 0.50 to 0.91). In the Brainspan and PsychENCODE datasets, comparisons had consistent effect sizes when averaging across individuals of all ages rather than just adults (Dataset S2). In the GTEx sample, the effect sizes remained stable whether or not including individuals over 60 or including individuals with evidence for brain disease or other issues that might confound transcriptomic data (Dataset S2).

### Whole-Brain Spatial Gradients Capture the Patterning of Biological Features across the Cerebral Cortex.

**Fig. 2.**
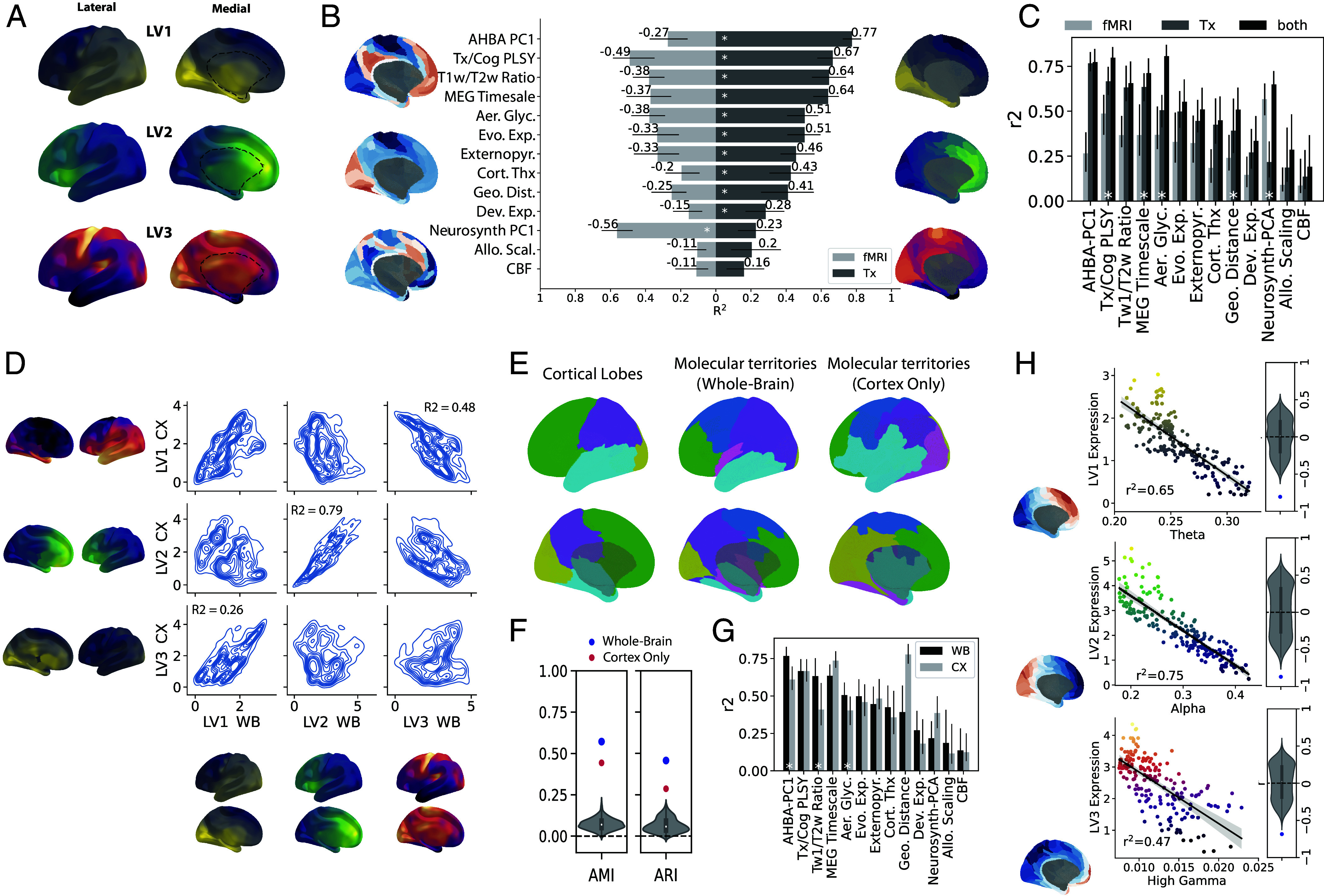
Cortical interaction of whole-brain transcriptomic gradients outlines fundamental properties of brain organization and function. Molecular gradients are spatially informative of the topography of diverse brain properties and interact to form distinct functional territories. (*A*) From *Top* to *Bottom*, a cortico-subcortical projection of PLS LV1, LV2, and LV3. (*B*) PLS latent variables outperform fMRI gradients in predicting brain features. Each of the three PLS LVs, as well as the top three principal fMRI gradients from Margulies et al. (20), were parcellated using the Glasser atlas. For several brain features (*SI Appendix*, *Materials and Methods* and Dataset S3), a linear model was fitted with the feature as the dependent variable and either the PLS LV (red) or fMRI principal gradient values (blue) as independent variables. Bars indicate the total model R^2^ (explained variance). CIs were obtained using bootstrapping. White stars indicate one model R^2^ was significantly larger than the other using bootstrap tests. (*C*) Linear models were fit for the same features in (*B*) with the feature as the dependent variable and either the three spatiomolecular axis values (from PLS, blue), the three fMRI principal gradient values (red) or all six variables as predictors (teal). Bars indicate the total model R^2^ (explained variance). CIs were obtained using bootstrapping. White stars indicate variables for which models incorporating both feature sets explained significantly more variance than models with only one feature set. (*D*) Cortical gradients were derived by fitting a new PLS model cross-decomposing gene expression and space, but this time using only cortical tissue samples. The three cortical gradients were projected to a cortical surface, and this surface projection was compared to the cortical projection of the whole-brain gradients used throughout this study. The comparison is made via vertex-wise spatial correlation, and the relationship is visualized using aerial density plots where higher “altitude” represents a greater density of samples. (*E*) Cortical vertices were clustered to identify six molecular territories of overlapping spatiomolecular gradient concentrations (*SI Appendix*, Fig. S7 *A* and *B*). These molecular territories (*Center*) were compared to (*Left*) a surface rendering of lobar regions of interest, representing the occipital (yellow), parietal (purple), frontal (green), and temporal (turquoise) lobes, and molecular territories created using the cortex-only gradients (from *D; Right*). (*F*) Adjusted mutual information (AMI) and adjusted Rand index (ARI) were used to compare the two molecular cortical maps to the vertexwise canonical lobar map. The blue (whole-brain) and red (cortex-only) dots represent the values, while the null distributions were created by using these two metrics to compare the lobar map to 1,000 spun versions of the molecular territories. (*G*) Using bootstrap tests, the spatial distribution of several cortical features was better explained by whole-brain gradients than by cortical gradients (indicated with white stars). (*H*) Maps of cortical activity at different wavelengths acquired from MEG demonstrate topographic correlation to molecular gradients. Spatiomolecular axes and MEG maps were parcellated with the Glasser atlas and compared. The strongest correlation for each gradient is shown. Each dot represents a region; brains next to each graph represent cortical renderings of the respective MEG map. Beside each plot, the observed *r*-value of the correlation is compared to a null distribution of correlations created between the MEG map and 1,000 permutations of the gradient maps generated using a conservative permutation procedure that preserves the spatial covariance structure.

The above findings led us to speculate that whole-brain spatial gradients may be associated with other fundamental properties of cortical organization. One common way to understand cortical functional organization is through primary gradients of functional connectivity variance derived from fMRI, which are thought to define multiple cortical functional hierarchies (20). We directly compared the explanatory power of functional connectivity gradients to that of our spatial transcriptomic gradients by building models composed of each of these gradients to predict several brain features (Fig. 2 *A*–*C*). We found that molecular cortical gradients (Fig. 2*A*; see *SI Appendix*, Fig. S2 for further disambiguation of cortical projection) explained significantly more variance than functional gradients in eight of 11 brain features assessed, including cortical thickness and myelination, aerobic glycolysis, and allometric scaling (Fig. 2*B* and *SI Appendix*, Fig. S6). In contrast, functional gradients explained more variance than the molecular gradients only in meta-analytic functional coactivation patterns. We next conducted models using both types of gradients to discern whether the molecular and functional gradients were contributing overlapping or distinct information to the spatial topography of cortical features (Fig. 2*C* and *SI Appendix*, Fig. S6). Models for five variables (aerobic glycolysis, geometric distance, principal meta-analytic functional gradient, intrinsic timescale, and principal gene-related functional gradient) explained significantly more variance when both types of gradients were included, indicating that functional and molecular modalities contributed complementary information. This was most striking for aerobic glycolysis, for which 75% of the variance in spatial topography could be explained by the combined gradients. These analyses together delineate a strong relationship between variation in fundamental properties of brain organization and the distribution of spatiomolecular gradients.

Given the causal role of interacting gene expression gradients in the developmental process of arealization, we hypothesized that cortical overlap of whole-brain spatiomolecular gradients may be involved in macroscale organization of regions with distinct areal boundaries. To evaluate this, we performed a clustering analysis to identify distinct territories of spatiomolecular gradient that overlap in the cerebral cortex (*SI Appendix*, Fig. S7*A*), which converged on six distinct territories (Fig. 2*E* and *SI Appendix*, Fig. S7*B*). The spatial extent of the territories overlapped with established functional and cytoarchitectonic subdivisions of the cerebral cortex: Territory 1 (blue) outlined motor and premotor cortex, Territory 2 (green) covered the prefrontal cortex, Territory 3 (purple) included parietal and lateral occipital cortical areas, Territory 4 (pink) subsumed limbic and subcortical regions, Territory 5 (yellow) outlined primary visual cortex, and Territory 6 (teal) encompassed the temporal lobe (Fig. 2*E* and *SI Appendix*, Fig. S7*B*). The molecular territories were compared directly to a lobar parcellation of the cortex, and the two parcellations showed good agreement (adjusted mutual information score = 0.57, p_spin_ < 0.001, using 1,000 spatially preserving permutations; adjusted Rand index = 0.46, p_spin_ < 0.001; Fig. 2*F*). To further explore whether these territories indeed represent functional divisions of the telencephalon, we used meta-analytic functional coactivation data (47) to examine the behaviors associated with activation of regions within each territory (*SI Appendix*, Fig. S7*C*). This analysis recapitulated well-established structure–function relationships in the brain. Namely, the six cortical territories were differentially involved in tasks and movement (Territory 1; blue), social cognition (2; green), spatial orientation (3; purple), valuation and encoding (4; pink), visual function (5; yellow), and language (6; teal) (*SI Appendix*, Fig. S7*C*).

Finally, we wished to directly test the hypothesis that whole-brain gradients could model cortical organization above and beyond cortical gradients. We found that LVs generated from only cortical tissue samples differ somewhat from LVs generated from all tissue samples, sharing 26 to 79% of variance in spatial distribution (Fig. 2*D*). Importantly, both qualitative (Fig. 2*E*) and quantitative (Fig. 2*F*) analysis showed that molecular territories generated with only cortical samples resembled the canonical lobes of the brain less than molecular territories generated using all brain samples. Finally, whole-brain LVs better explained the regional cortical distribution of myelination (T1/T2 ratio), aerobic glycolysis, and the first principal component of gene expression than cortical-only LVs, where cortical-only LVs did not better explain any measured brain feature compared to whole-brain LVs (Fig. 2*G*). Taken together, these results suggest that molecular gradients of the cerebral cortex may in part reflect cortical representations of whole-brain gradients.

### Candidate Functional Brain Phenomena Underlying Molecular Gradients.

The previous analyses revealed that spatiomolecular gradients are in part spatially differentiable from the canonical functional gradients of the cerebral cortex. Yet, given that the interaction of molecular gradients gives rise to functionally distinct territories, we expected to find some evidence for differences in modes of functional communication along molecular gradients. Given previous literature has described traveling waves of cortical activity propagating across the cerebral cortex (48–50), we were curious whether these oscillations aligned with spatiomolecular gradients. We correlated spatial patterns of intrinsic spectral content of mass neuronal activity across several different wavelengths (measured using magnetoencephalography; MEG) with the three PLS latent variables. We found that each component strongly matched the spatial oscillation pattern of a different wavelength (Fig. 2*H* and *SI Appendix*, Fig. S8). LV1 matched theta activity (r = −0.81, p_spin_ < 0.001), LV2 matched alpha activity (r = −0.87, p_spin_ < 0.001) and LV3 best matched high gamma activity (r = −0.69, p_spin_ < 0.001). These results suggest that major components of subsecond neuronal communication occur preferentially along paths defined by spatiomolecular gradients.

### Spatiomolecular Gradients Are Differentially Conserved across Phylogeny.

**Fig. 3.**
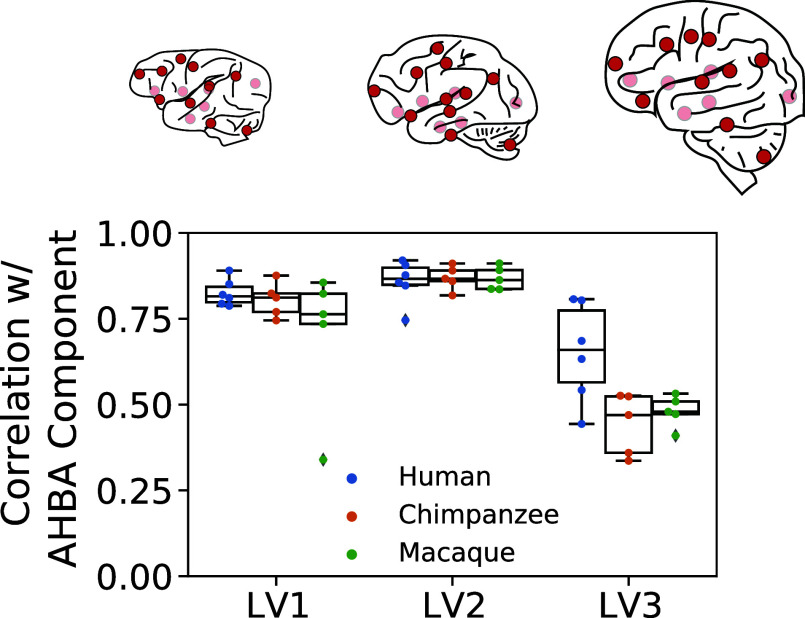
Tracking spatiomolecular gradients across phylogeny. The PLS latent variables are replicable across primate species. Six adult human, five adult chimpanzee, and five adult macaque brains were available from the PsychENCODE dataset with tissue samples extracted from the same 16 brain regions (indicated with red dots). Component correlations (as described in Fig. 1*I*) for each component were generated for each individual (see *SI Appendix*, Fig. S9 for individual null models). There was a significant component by species interaction, where LV3 expression was greater in humans than in other primates.

Previous analyses showed remarkable consistency in the expression of specific spatiomolecular gradients across several different datasets. Given extensive prior evidence linking spatial variation in expression across species and human development (37, 51), we sought to evaluate whether spatiomolecular gradients were emergent (uniquely human) features of the brain, or whether they represent phylogenetically conserved features observable across the brains of other primate species. To evaluate this, we evaluated the expression of the three LVs using tissue samples extracted from adult macaque, chimpanzee, and a separate set of human brains (Fig. 3). Using the same approach described above (Fig. 1*I*), we tested how well the individual regional expression pattern of each LV matched the regional expression pattern of the discovery LVs compared to null models (Fig. 3). We found regional gradient expression to replicate well across humans (LV1 r = 0.90 to 0.98; LV2 r = 0.81 to 0.92; LV3 r = 0.71 to 0.85), chimpanzees (LV1 r = 0.92 to 0.96; LV2 r = 0.80 to 0.93; LV3 r = 0.46 to 0.72), and macaques (LV1 r = 0.91 to 0.94; LV2 r = 0.85 to 0.93; LV3 r = 0.60 to 0.77). These individual correlations with the discovery dataset exceeded chance (*P* < 0.05) for all individuals, except for one chimpanzee individual for LV3 (*SI Appendix*, Fig. S9*B*). A two-way ANOVA showed a significant species by component interaction (F[4;39] = 3.43, *P* = 0.017), where LV3 similarity was higher in humans compared to other primates. This suggests that LV3—which notably was the most variable across the cortex (*SI Appendix*, Fig. S4*A*)—is less conserved across primate species compared to LV1 and LV2. We further show that the similarity of the LVs across species was not simply a product of generally conserved brain gene expression patterns across the three species (*SI Appendix*, *Results* and Fig. S9*A*). In all, we found strong evidence for the existence of homologous LV1 and LV2 gradients in chimpanzees and macaques, indicating conservation of these organizational phenomena at least across primate species.

Finally, we wished to assess whether the molecular gradients seen in human and primate brains were also present in mouse brains. The PLS model fitted to human gene expression data was applied to a dataset of whole-brain postnatal mouse gene expression. The model, based on human gene expression, predicted the location of mouse tissue samples (*SI Appendix*, Fig. S10*A*). While explaining substantially less variance in spatial location compared to the human dataset (Fig. 1*E*), the model predictions nonetheless greatly exceed chance (all *P* < 0.001) and were best for predicting location along the y coordinate. When projecting the human LVs onto the mouse brain, the distribution of LV1 and LV2 resembled that of humans (*SI Appendix*, Fig. S10*B*). LV1 showed a rostral-caudal pattern moving from brainstem and cerebellar regions toward the cerebral (and olfactory) cortex, while LV2 showed a largely anterior–posterior pattern. Genes driving these two patterns in the mouse were cross-referenced with top gradient-related genes in humans (see below; Fig. 5*A*). For LV1, this set of genes list included transcription factors and developmental patterning genes (*PAX6*, *FOXG1*), genes with known area-specific and cell-type specific expression (*CALB2*, *ZIC1*, *GDF10*), and other genes involved in neurodevelopment (*GDA*). The gene list for LV2 included *NEFH*, *RELN*, and *FOXF2*. In contrast to the other two gradients, the LV3 pattern was largely unfamiliar, with positive peaks in the dorsal brainstem, hippocampus, and thalamus and negative peaks in the olfactory bulb, ventral midbrain, and pons.

### Differential Maturation of Gradients Observable across Species.

**Fig. 4.**
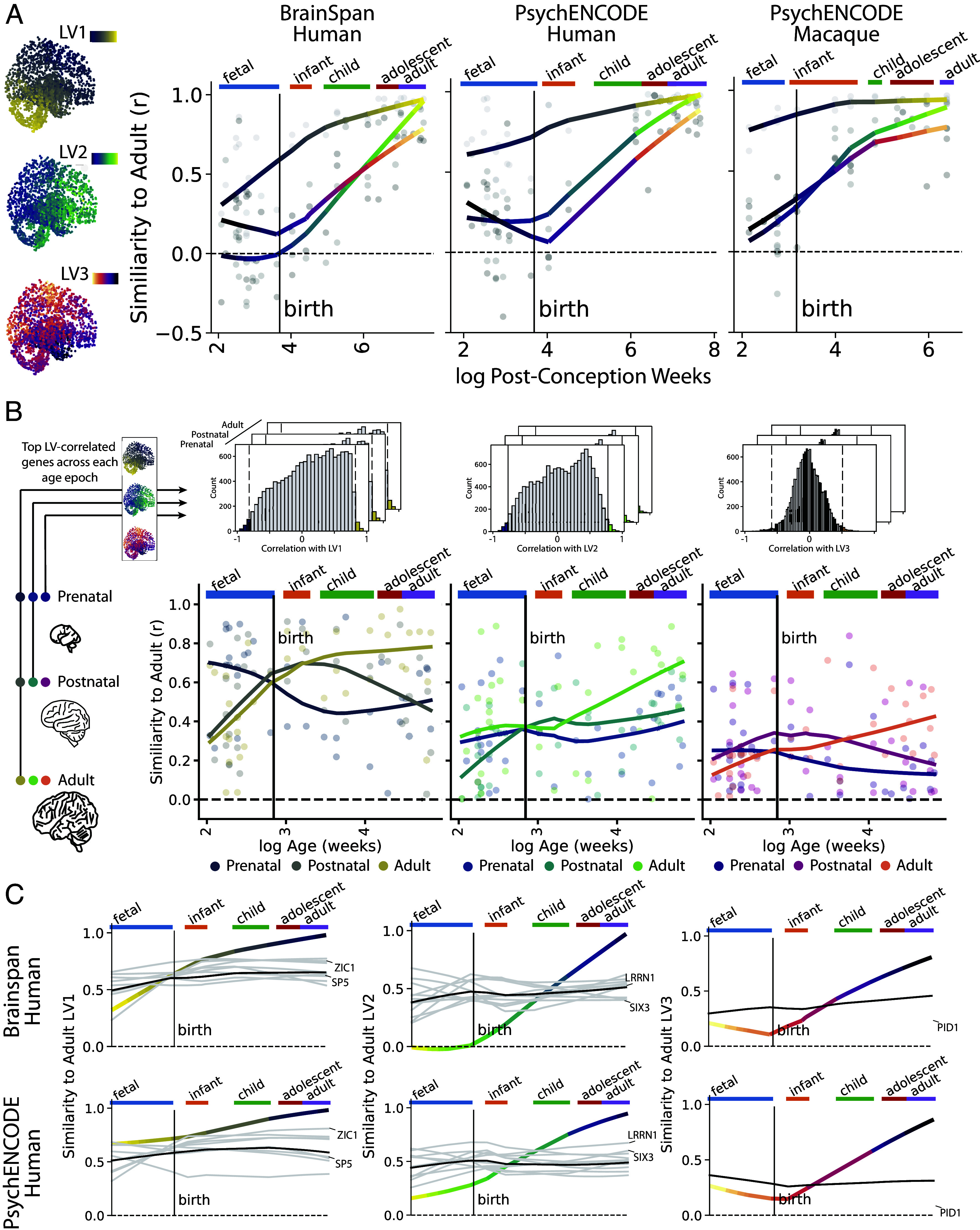
Tracking spatiomolecular gradients across ontogeny. The PLS latent variables show distinct and replicable lifespan developmental trajectories determined by both phasic and constant gene expression patterns. (*A*) The method described in Fig. 1*I* was applied to data from individual brain donors across the development spectrum in the BrainSpan dataset (*Left*) and PsychENCODE human dataset (*Center*) and the PsychENCODE macaque dataset (*Right*). Plots represent the relationship between log Age (in postconception weeks) and individual-level component correlations with AHBA (adult) PLS latent variables. Developmental gradient expression is remarkably similar across datasets, with adult-like LV1 expression present early in development, and adult-like LV2 and LV3 expression accelerating after birth. (*B*) Gradient-associated genes were established at each developmental epoch (prenatal, postnatal, adult) for each LV by finding genes with the highest regional correlation (top 1%) with adult regional expression of that LV. This resulted in three sets of gradient-associated genes for each LV: prenatal (dark colors), postnatal (middle colors), and adult (light colors). Similarity to adult expression was then plotted across the lifespan for each gene set. While most evident for LV1, the general trend showed prenatal gradient-associated gene expression declining after birth, adult expression rising throughout development, and postnatal gene expression peaking just after birth. (*C*) Clustering was used to identify a set of individual genes that demonstrate high regional expression similarity to adult LVs throughout brain development (i.e., are nontransitional). For all three LVs (columns) and both human datasets (rows), the y-axis represents regional similarity to adult AHBA LV expression. The trajectories of individual genes falling into the nontransitional clusters across both datasets are visualized juxtaposed against the trajectory of LVs. Showing high similarity to adult LV expression during prenatal development and maintaining this expression level into adulthood, these genes are candidates for establishment and maintenance of the LVs. A few example genes are highlighted to demonstrate consistency in gene trajectories across datasets. All candidate genes are listed in *SI Appendix*, Fig. S12.

The directional radiation of the three spatiomolecular gradients resembles the morphogen diffusion patterns of whole-brain developmental spatial gradients (Fig. 1*A*). Therefore, we wished to assess whether the molecular gradients identified in our adult samples are present during fetal brain development and how they develop over the lifespan. We used expression patterns of genes shared across datasets to examine the development of the PLS LVs across two developing human datasets spanning postconception week 8 to postnatal year 40, and one developing macaque dataset spanning postconception week 8 to postnatal year 11. Specifically, we compared each individual’s PLS LV expression to that of the adult discovery set using regional correlations (from Fig. 1*I*). Expression of adult-like gradients varied systematically with age in a gradient-dependent fashion (Fig. 4*A* and *SI Appendix*, Figs. S11*A* and S12). Supporting previous findings (37), across all components, variance in expression was highest prenatally (*SI Appendix*, Fig. S11*A*). The developmental trajectories of gradient expression were remarkably similar across datasets and across species. Across all three datasets, correlation with adult expression of the LV1 gradient was already nonzero at early measurement, and slowly became more adult-like with age. In contrast, LV2 and LV3 expression was not observable at birth but increased sharply throughout development, finally reaching adult-like levels at adulthood.

These results indicated the possibility that different genes are involved in the establishment, regulation, and expression of molecular gradients at different epochs of neurodevelopment, a concept supported by previous work (37). To test this hypothesis, we identified sets of genes with expression patterns resembling the adult LVs, but separately in prenatal, postnatal, and adult developmental epochs (Fig. 4*B*). A clear pattern emerged across LVs but was most prominent in LV1: LV-like expression of prenatal genes peaked prenatally and declined after birth, while LV-like expression of postnatal genes peaked just after birth, and LV-like expression of adult genes steadily increased over neurodevelopment (Fig. 4*B*). In all, different genes showed a gradient-like expression at different epochs, suggesting the possibility of different genes helping to consolidate or regulate these molecular gradients during different phases of neurodevelopment.

The previous analyses raise the question of whether there are any genes that show an LV-like expression pattern throughout development, possibly nominating them as important components in establishing and maintaining molecular gradients. Indeed, a subset of LV-associated genes showed an adult-like regional expression pattern throughout brain development, including during prenatal periods, across both human datasets (Fig. 4*C* and *SI Appendix*, Fig. S12). This set included genes known to be involved in areal patterning (e.g., *SIX3*, *SP5*, *ZIC1*). Most of these genes showed a similar developmental pattern in macaques as well, with a few notable exceptions (*SI Appendix*, Fig. S12).

**Fig. 5.**
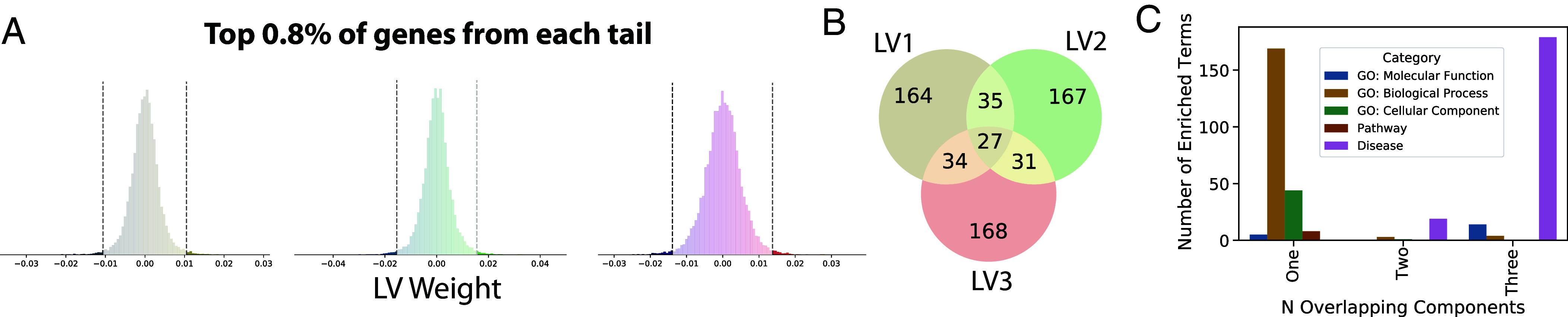
Annotation of gradient-associated genes. A core set of genes contributing to all three LVs are highly enriched for diseases. (*A*) Distributions of PLS LV weights across genes. Genes weighted in the top 0.8% (5% / two tails / three components) of either tail of this distribution (130 per tail) were selected for annotation. (*B*) Venn diagram showing how many genes were unique to one LV or shared among multiple LVs. (*C*) Gene set enrichment was executed on genes that were involved in one, two, or all three PLS latent variables. Genes belonging to only one component were mostly enriched for biological processes, whereas genes belonging to all three components were mostly enriched for diseases (*SI Appendix*, Fig. S13).

### Molecular Gradients Are Composed of Genes Relating to Neural Development and Disease.

Having established directional molecular gradients in the adult human brain, we were interested in understanding the roles of genes that contributed to them. We specifically focused on genes within the top 5% (adjusted for multiple comparisons; see *SI Appendix*, *Methods*) of each component (Fig. 5*A*). We found a clear and robust enrichment of terms relating to neural and neuronal development and morphogenesis, as well as synaptic signaling and extracellular matrix development (*SI Appendix*, Fig. S13*A* and Dataset S4). Furthermore, of 38 transcription factors that are known to be expressed in a gradient-like pattern in the developing mouse ventricular zone (6), 26% were included among our list of human gradient-associated genes (random gene set null mean = 3.3% [95% CI: 0.0 to 7.9%], p_[perm]_ = 0.001). This included some well-characterized transcription factors (e.g., *PAX6*, *SIX3*), as well as genes known to exhibit regionally specific or gradient-like expression in early development (*BCL11B*, *EPHA3*, *EPHA5*, *CDH6*, *NR2F2*, *PCDH8*, *SLN*, *TSHZ2*) and several members of the Wnt and cadherin/protocadherin family (Dataset S1). Interestingly, we also observed that multiple genes involved in gradient organization are known to be associated with neuropsychiatric disorders such as schizophrenia, autism, and bipolar disorder, demonstrating significant enrichment (*SI Appendix*, Fig. S13*B*). To confirm these associations, we assessed significant enrichment of genes from each gradient in a multivariate GWAS across psychiatric disorders (52). Genes in LV1 (*P* = 0.026) and LV3 (*P* = 0.0091) were enriched in factor one of this GWAS, which was associated with psychosis, mania, and depressive disorders. No enrichment was found for the second factor, which was composed of schizophrenia and bipolar I disorders (*SI Appendix*, Fig. S13*C*).

We next examined whether any overlap existed in the genetic anatomy of each LV. Despite the fact that the PLS approach enforces orthogonality among its LVs, we found that several genes contributed to more than one gradient, and 27 genes contributed to all three (Fig. 5*B*). Interestingly, genes that contributed to only one gradient tended to be associated with specific biological processes but not diseases, whereas genes involved in all three components were associated with diverse neurological, neuropsychiatric, and nonneural diseases but with few biological processes (Fig. 5*C* and Dataset S5). Three of the genes highlighted earlier as candidates for early gradient establishment (*HRH3*, *TLL1*, *MGAT4C*) were also present in this set of multigradient, disease-associated genes.

Finally, in order to gain a better understanding of direct relationships between brain gene expression and a broader array of relevant phenotypes, we leveraged a recently published phenotype-wide association study (PheWAS) using brain expression-based multiancestry meta-analytic quantitative association loci (mm-QTLs) (53). Specifically, we identified single nucleotide polymorphisms (SNPs) that were significantly associated with expression of LV-associated genes in the brain, and which also showed genome-wide significant associations with biological traits and brain-related phenotypes (*SI Appendix*, Fig. S13*D* and Dataset S6). Many SNPs associated with brain expression of gradient genes were also associated with body size and distribution, metabolism, and circulation. However, additional brain-related associations were noted, including amyotrophic lateral sclerosis (ALS) in LV1, sleep and risk-taking behavior in LV2, and educational attainment and cognitive traits in LV3.

## Discussion

Directional morphogenetic gradients are a well-established organizational element of the developing brain, serving as scaffolds for the coordination of functional arealization. We demonstrate multiple lines of associational evidence that these gradients are retained in the adult human brain, in the form of robust and systematic transcriptional variation along three spatially embedded brain axes. These spatiomolecular gradients traverse the cerebral cortex along paths of intrinsic neural oscillations, and the interaction of the gradients delineate territories representing well-described anatomical and functional cortical divisions. In all, we describe a reproducible pattern of brain gene expression that resembles developmental morphogen gradients, and which interacts in the cerebral cortex to form functionally differentiated regions in the adult brain. These results raise the possibility that the spatiomolecular gradients we describe may be echoes of fundamental organizational axes of brain functional organization, coordinated prenatally through morphogen diffusion and consolidated throughout development.

Functional gradients in the adult human cerebral cortex have been a topic of concerted study in recent years, where a sensorimotor–association axis has been proposed as a major feature of functional organization that can also explain variance in many aspects of cortical physiology and neurobiology (20, 21). In parallel, the introduction of transcriptomic datasets with dense cortical sampling has allowed the establishment of intrinsic components of cortical gene expression (23, 33, 35, 54). Several studies have further shown a spatial correspondence between these principal axes of function and gene expression (26, 55–57). Our approach differed from these previous studies in two critical ways: i) We used data from the entire brain rather than only the cerebral cortex; and ii) we enforced a degree of supervision by constraining transcriptomic covariation to directional radiation, in accordance with the distribution of theoretical developmental gradients (which are embedded in Euclidean space). The resulting transcriptomic gradients bore some spatial resemblance to fMRI-based functional gradients, but were far more similar to patterns of neural oscillations measured at millisecond timescales. These findings corroborate previous studies finding strong spatial relationships between cortical gene expression and millisecond timescale functional activity (25). This is notable given that electrophysiological and fMRI measure brain activity at different time scales, overlapping mostly at the high-frequency band (58–62). Together, these results suggest that our spatiomolecular gradients and previously described functional gradients probably represent two distinct elements of cortical organization. As such, we hypothesize that spatiomolecular gradients dynamically coordinate physiological properties of the brain, and their confluent cortical topographies enforce the distribution of functionally distinct regions in space. In contrast, we speculate that the primary functional gradients help to coordinate optimal communication between these spatially distinct regions, forming networks of long-range projections that likely manifest as functional networks measurable with fMRI (57, 63–66). Both organizational elements are likely reflected by independent functional activity at different timescales (67, 68), a notion supported by our finding that functional and spatiomolecular gradients each contributed additive information in explaining variance in the spatial distribution of brain energy consumption (aerobic glycolysis). This idea is also supported by recent charting of functional organization in the brain, where prenatal functional organization is reminiscent of the molecular territories described here (69), and by the recent finding that functional organization itself may be fundamentally geometric (70).

The spatial axes along which these gradients vary are phylogenetically ancient. Rostral-caudal and dorsal-ventral identity are evolutionarily conserved aspects of body-plan formation that are genetically encoded (9, 71, 72). Many similar signaling pathways also encode these spatial axes in the brain to create explicit divisions (9). Some examples of this organizational feature include the fact that phylogenetically ancient species exhibit explicit structural divisions separating brain segments along the rostral-caudal axis (73), that cell lineage studies find biased allocation along rostral-caudal brain axes in early development (74), and that cerebral organoids spontaneously self-organize around spatial axes (11). Therefore, it is unsurprising to find excellent reproducibility of the human-derived rostral-caudal (LV1) and dorsal-ventral (LV2) spatiomolecular axes in various nonhuman primate species and evidence for these gradients in mice as well. In contrast, LV3 showed the most variation within the cerebral cortex, a brain region of disproportionate size in primates, and in humans compared to other primates (46). Interestingly, this gradient was not as reproducible in nonhuman primates, was not evident in mice, was made up of genes enriched for human neuropsychiatric disorders and associated with educational attainment and cognition, and was expressed in a pattern similar to gamma waves observed during complex cognition (75). This component also continued to consolidate throughout childhood and adolescence and demonstrated few genes with adult-like expression during early development, as opposed to LV1, which showed a fairly adult-like distribution already during early life. Together, these findings outline how various molecular gradients interact during different epochs of brain growth to guide regional differentiation and functional specialization.

Previous studies suggest that cortical expansion can allow greater cortical differentiation, particularly along spatial axes (76) similar to those characterized by our PLS analysis (77, 78). Therefore, a gradient organizational framework can create an intimate relationship between (brain) compartment size or shape and the complexity of regional differentiation (79). This may be an important principle underlying the observation that specialized function co-occurs with nonlinear variation in brain morphology, even among conspecifics (80). This also may explain the finding that LV3 was less reproducible in other primate species with less expanded cortex. However, the directionality of the relationship between brain shape/size and gradient expression is still not clear. We found that many of the gradient-associated genes in this study showed pleiotropic effects on human physical form and function. This finding supports the observation that genes regulating brain axes may play a similar role on the axes of other organs. In addition, in a scenario where organ size relates to expression of gradient-forming genes, coexpression of genes supporting the attainment and/or maintenance of larger size may also be expected. This may explain the observation that mutations within some of the LV-associated genes in our study are linked to interindividual variation in metabolic and anthropometric traits.

Several genetic studies also support the link between gradients and brain organization. The advent of large neuroimaging–genetics biobanks has brought about unprecedented power for detecting genetic variants that relate to brain structure (81–87). A common finding across several of these papers (40, 41, 84, 85) is that correlation in genetic determinants of morphometric features across brain regions varies. Interestingly, these studies each show that this variation forms gradients across the cortex, and those gradients almost perfectly overlap with the transcriptomic gradients described presently. These studies, in combination, provide convergent genetic and transcriptomic evidence that gradients are key facets of cortical organization. There is great potential for future investigations to bridge these studies by establishing causal links between key genetic mutations identified in these studies and transcriptomic changes described in this paper. Similarly, transcriptome-wide association studies (TWAS) attempt to contextualize genome-wide associations with more informative, tissue-specific profiles, capitalizing on the cis-regulatory component of gene expression. Future work directly examining the relationship between these gradients and functional outcomes and disease-related traits in large-scale TWAS would be important to pursue.

The role of morphogen gradients in the coordination of downstream molecular events during neurodevelopment is well established (1, 2). We show that these molecular trails are represented in the adult brain by patterns of genes coexpressed along similar axes. The genes composing these molecular axes included many known developmental morphogens and transcription factors and were enriched almost exclusively for biological processes relating to neural organ and neural cell development. On the other hand, the molecular axes were not reliably observed in prenatal brains, and instead only became maximally recognizable in adulthood, potentially due to differential contributions from functionally related genes. Previous literature has described waves of transcriptomic changes that occur along stereotyped developmental schedules (37, 88, 89). As tissue samples used to discover these molecular gradients were sourced exclusively from adult donors, the resulting gradients may represent a confluence of multiple waves of transcriptional change consolidating the axes that begin to be established during early development—a so-called “palimpsest” model as described previously (90). Supporting this theory, we found many genes were expressed in gradient-like patterns only during certain phases of development. However, we also found that a subset of the top gradient-associated genes for LV1 and LV2 demonstrated gradient-like expression patterns prenatally and throughout development. Some of these genes may be involved in the early establishment and/or long-term maintenance of spatiomolecular gradients, which are otherwise consolidated across development by the recruitment of many other genes with differing functional consequences.

Our analyses suggest that spatiomolecular gradients are highly conserved across adult individuals, underscoring the importance of both the establishment and maintenance of these gradients for healthy brain functioning. Previous literature suggests disruption of the formation of early developmental gradients through mutations to gradient-associated genes can cause mortality and severe developmental disorders (12). Our analysis supports this premise, as a small set of genes contributing to all three adult brain gradients were strongly enriched for disease associations. These genes may play an important role in anchoring one or more gradients, and their disruption could impact morbidity through complex perturbation across multiple components of brain organization. We furthermore found that genes associated with adult LVs are enriched for several psychiatric disorders and demonstrated overlap with a multifactorial psychiatric disease GWAS (52). In most cases, the underlying link between gene and disease is not known, so it is difficult to ascertain whether spatial patterning could play a direct role. However, several of the genes in this list of disease-enriched have well-described roles in spatial patterning [e.g., *GAL* (91), *WNT10A* (92), *TLL1* (93), for example]. Others have been directly implicated in congenital malformations associated with cognitive impairment [e.g., *PRRX1* (94), *NR2F2* (95), *MGAT5* (96)]. In most of these latter cases, direct links between the phenotype and neurodevelopmental patterning have not yet been investigated, but one recent study showed *MGAT5* knockout resulted in a spatial shift of cortical neuron layers (97). In other cases [e.g., *MET* (98), *TLL1* (99)], genetic knockouts result in death due to malformations of other organs. This may indicate a role of these genes in patterning outside of the brain but may also preclude examination of how brain development (which occurs later) might be affected. There are also several genes that are involved in cognitive or neuropsychiatric disorders, such as *MGAT5* (96), *NRXN1* (100), *HRH3* (101), and *CDH13* (102, 103). It is tempting to speculate that this could be driven by impaired spatial patterning during development, especially since two of these genes (*HRH3*, *MGAT5*) were also nominated as candidate regulators of the LVs in this study. Experiments must first be performed to validate this hypothesis, but our study suggests that molecular gradients may be important elements in the emergence of brain-related traits.

There are several potential limitations that must be acknowledged while interpreting the present findings—the foremost of which is the reliance on whole-brain spatial correlations to draw inference. While the comparison of densely sampled, multimodal whole-brain information is a distinct strength of our approach, correlations of the spatial topography of two signals, no matter how high they may be, are not indicative of complex relationships. We tried to mitigate this limitation by using analysis-specific permutation tests and spatially aware null models to provide conservative testing for significant relationships. Still, there are no experimental manipulations in this study, and we therefore cannot establish causality. Many state-of-the-art null modeling approaches in neuroimaging seek to correct for spatial autocorrelation (104), whereas this was the phenotype of interest in the present study. We overcame this limitation by validating our molecular gradients in several independent datasets, albeit these datasets had much more limited spatial sampling of the brain. Bioinformatic annotation of gene sets also comes with numerous limitations. Transcriptomic information does not represent protein concentration with great fidelity (105), and relationships between gene sets and enriched terms do not entail a causal relationship between our gradients and biological processes or diseases. We addressed this limitation by validating disease associations with different techniques and by identifying phenotypes relating to SNPs that were themselves associated with brain gene expression of LV-associated genes. However, each of these approaches is not without its own set of limitations. Finally, we attempt to further protect against other limitations of data analysis by publishing all code used to analyze the present data in order to maximize reproducibility.

This study establishes directional gene expression gradients as measurable phenomena in the adult human brain and highlights their potential as possibly key features of functional organization in the cerebral cortex. While many contemporary studies consider autocorrelated gene expression information an artifact or nuisance to be statistically removed (106), the role of this kind of signal must be acknowledged as a biological phenomenon with an important role in brain organization. Indeed, gene expression gradients are present across many organs (107, 108), likely due to their capability of optimizing tissue differentiation and enforcing organizational features such as symmetry and functional distribution. Future work should strive to characterize the developmental sequences of these gradients with greater temporal granularity and further demonstrate how variations in this process influence the brain and behavior at the individual level.

## Materials and Methods

Please see *SI Appendix* for a full description of the Methods. All analyses in this manuscript can be reproduced using code at https://github.com/PennLINC/Vogel_PLS_Tx-Space.

### Samples.

Data in this study included transcriptomic sequencing of tissue samples extracted from human, nonhuman primate, and mouse brains sourced from one discovery dataset (AHBA) and multiple external datasets (GTEx, PsychENCODE, BrainSpan). Samples were accompanied by either precise stereotaxic coordinates of extracted tissue, or information as to broad anatomical location.

### Derivation of Transcriptomic Gradients.

Our objective was to identify patterns of autocorrelated brain gene expression that vary systematically along a 3-dimensional linear axis across the whole brain (i.e., not just the cerebral cortex). We used PLS to determine modes of latent covariance between gene expression and euclidean space (*SI Appendix*, Fig. S1). Robustness was assessed using k-fold cross-validation, out-of-sample prediction, and spatial permutation tests.

### Cortical Surface Analysis.

PLS LVs were interpolated over a cortical surface, and their spatial distribution was compared to that of other cortical properties (Dataset S3) using bootstrapped linear models. The strength of these associations was compared to the same models built using the top three functional gradients (20). Cortical molecular territories were derived by clustering all three LVs (*SI Appendix*, Fig. S7*A*). These territories were annotated using neurosynth meta-analytic functional decoding (47) and were spatially compared to cortical lobar divisions using mutual information and Rand index. This latter comparison was assessed against cortical territories created using only cortical tissue samples (as opposed to cortical territories using whole-brain tissue samples).

### Expression of Gradients across Development and Species.

The PLS model was used to derive LVs at the individual level of external datasets (Fig. 1*I*). Individual-level out-of-sample LV expression was spatially correlated to group-level LVs from the discovery dataset (which was composed of adult humans). This generated an r-value which we used as a measure of similarity of individual LV expression to normative adult human LV expression. This similarity measure was compared across humans, chimpanzees, and macaques from external datasets and was also assessed across log age in two human and one macaque external datasets.

### Annotation of Gradient-Associated Genes.

Genes showing expression patterns most similar to each LV were aggregated across several developmental epochs. Genes are described that maintained LV-like expression across neurodevelopment across multiple human datasets, and these genes were also validated in macaques. Next, a list of genes was curated showing strong loading on each LV in the discovery dataset. Enrichment of these gene lists for biological processes, molecular functions, cellular components, phenotypes, and diseases was assessed using ToppGene (109), and enrichment for previously described prenatal morphogens was also assessed (6). MAGMA (110) was further used to assess enrichment of genes that are associated with psychiatric disorder through GWAS (52). Finally, putative phenotypic associations of LVs were assessed through a phenome-wide association based on multiancestry meta-analytic quantitative trait loci (53).

## Supplementary Material

Appendix 01 (PDF)

Dataset S01 (CSV)

Dataset S02 (CSV)

Dataset S03 (CSV)

Dataset S04 (CSV)

Dataset S05 (CSV)

Dataset S06 (CSV)

Dataset S07 (CSV)

Dataset S08 (CSV)

## Data Availability

Previously published data were used for this work (17, 47, 51–53, 111–113).
