# Supplementary material for: Deciphering the functional specialization of whole-brain spatiomolecular gradients in the adult brain
Source: Proc Natl Acad Sci U S A. 2024 Jun 11;121(25):e2219137121. doi: 10.1073/pnas.2219137121 (PMC11194492; doi:10.1073/pnas.2219137121)
Supplement: Supplementary file 1 — Appendix 01 (PDF) [file pnas.2219137121.sapp.pdf]

Supporting Information for "Deciphering the functional specialization of whole-brain spatiomolecular gradients in the adult brain"

Jacob W Vogel*[1,2,3], Aaron Alexander-Bloch[3,4,5], Konrad Wagstyl[6], Maxwell Bertolero[2,3], Ross Markello[7], Adam Pines[2,3], Valerie J Sydnor[2,3], Alex Diaz-Papkovich[8,9], Justine Hansen[7], Alan C Evans[7], Boris Bernhardt[7], Bratislav Misic[7], Theodore Satterthwaite[2,3,#], Jakob Seidlitz[3,4,5#]

[1] Department of Clinical Sciences Malmö, SciLifeLab, Lund University, Lund, Sweden
[2] Lifespan Informatics & Neuroimaging Center, University of Pennsylvania, Philadelphia, PA 19104
[3] Department of Psychiatry, University of Pennsylvania, Philadelphia, PA 19104
[4] Department of Child and Adolescent Psychiatry and Behavioral Science, The Children's Hospital of Philadelphia, Philadelphia, PA 19104
[5] Lifespan Brain Institute, The Children's Hospital of Philadelphia & University of Pennsylvania, Philadelphia, PA 19104
[6] Wellcome Centre for Human Neuroimaging, Institute of Neurology, University College London, WC1N 3AR
[7] McConnell Brain Imaging Centre, Montreal Neurological Institute, McGill University, Montreal, QC H3A 2B4, Canada
[8] Quantitative Life Sciences, McGill University, Montreal, QC, H3A 1E3
[9] McGill Genome Centre, McGill University, Montreal, QC, H3A 0G1

* Corresponding author – jacobwvogel@gmail.com
[#] Contributed equally as senior authors

SI Methods

All statistical analyses were performed and plots generated using the python programming language (v. 3.7.3), mainly using the numpy, scipy, pandas, sklearn, statsmodels and seaborn libraries. Code and library versions necessary to reproduce all analyses described can be found at https://github.com/PennLINC/Vogel_PLS_Tx-Space.

Samples and Preprocessing

Discovery Sample. The genomic gradients described and analyzed throughout the present study were generated using data from the Allen Human Brain Atlas. A detailed description of this dataset can be found elsewhere (1). Briefly, the dataset consists of 3702 tissue samples extracted from eight cerebral hemispheres across six human donors (1 female, ages 24.0-57.0, mean=42.50 +/- 13.38). Stereotaxic coordinates for each tissue sample were meticulously recorded by the anatomist during sample extraction, and these stereotaxic coordinates were transformed into a standard neuroimaging (MNI) reference space, available in the public Allen Brain Atlas dataset. The MNI coordinates were later updated to reflect non-linear transformation from donor to standard space (see below), and these coordinates were used to make the three-dimensional Euclidean coordinate matrix used in analysis. Each tissue sample underwent high throughput microarray mRNA analysis across 58,692 probes and underwent preprocessing as previously described (2, 3). The 58,692 x 3702 data matrix downloaded from (https://human.brain-map.org/static/download) was further processed using the abagen toolbox (version 0.1.3; https://github.com/rmarkello/abagen) (3) based on prior recommendations (2). Rather than averaging tissue samples within regions of a brain atlas, all samples were extracted and underwent preprocessing. The following text was generated directly from abagen, describing the exact methodologies employed during preprocessing:

First, microarray probes were reannotated using data provided by (2); probes not matched to a valid Entrez ID were discarded. Next, probes were filtered based on their expression intensity relative to background noise (4), such that probes with intensity less than the background in >=50% of samples across donors were discarded. When multiple probes indexed the expression of the same gene, we selected and used the probe with the most consistent pattern of regional variation across donors (i.e., differential stability; (4). The MNI coordinates of tissue samples were updated to those generated via non-linear registration using the Advanced Normalization Tools (ANTs; https://github.com/chrisfilo/alleninf). Inter-subject variation was addressed by normalizing tissue sample expression values across genes using a robust sigmoid function (5). Normalized expression values were then rescaled to the unit interval.

Finally, gene expression values were normalized across tissue samples using an identical procedure. Samples assigned to the same brain region were averaged separately for each donor and then across donors, yielding a regional expression matrix. After processing, a final 3466 sample x 15,634 gene matrix was used for subsequent analysis.

Human replication cohorts. Several publicly accessible datasets were used to replicate and extend results. The Brainspan "Developmental Transcriptome" dataset consists of 524 cortical, subcortical and cerebellar tissue samples extracted from the brains of 42 donors (19 female, ages 8 post-conception weeks to 40 years, mean=86 +/- 137 post-conception months). Each tissue sample underwent mRNA sequencing for 47,808 unique genes, and were preprocessed using methods previously described (6). This data was downloaded from https://www.brainspan.org/static/download.html in 2020. After removing duplicates, 13,750 genes were present in both the Allen Brain Atlas discovery dataset and the present Brainspan dataset, and this 524 sample x 13,750 gene dataset was used for analysis. To maximize consistency with the discovery datasets, initial reproducibility analyses were conducted only among the six adult donors in the Brainspan dataset (3 female, age 21-40, mean = 31.2 +/- 7.2).

A total of 2,483 cortical, subcortical, midbrain and cerebellar samples were extracted from the brains of 376 donors (105 females, age 20-75, mean = 58.7 +/- 9.7) from the NIH Genotype-Tissue Expression (GTEx) dataset Version 8(7). Each sample underwent mRNA sequencing for 15,758 unique genes. In order to obtain individual (rather than binned) ages, protected GTEx data was downloaded under accession number 26317. However, all other GTEx data besides age can be downloaded from (https://gtexportal.org/home/datasets). 12,647 genes overlapped with the discovery sample, and only data for these genes were used for subsequent analysis. After principal component analysis across technical variables, the first five principal components (PCs) were regressed from the expression data of each gene by finding the residual of an ordinary least squares regression model with expression data as the dependent variable and the five PCs as predictors. Previous work with the GTEx dataset describes removal of subjects based on presence of brain diseases and other potentially confounding factors (8). To rule these factors out as drivers of our results, we reproduced our results after subsampling the original 376 donors to only include 227 individuals included in (8), known to be free of brain diseases. Furthermore, to better match the age range of the initial discovery sample and to rule out advanced age as a drive of the results, initial reproducibility analyses were also repeated in both the original (n=178) and the subsampled (n=121) GTEx datasets after excluding individuals above the age of 60.

Non-human replication cohorts. To assess whether our results generalized across species, we leveraged a pre-curated dataset described in (9). This dataset combines tissue samples from the six adult humans, five adult chimpanzee brains and five adult macaque brains. The dataset can be downloaded from http://evolution.psychencode.org/#, where it is labeled as "Adult human, chimpanzee, macaque data" in the mRNA-seq tab. The dataset is pre-harmonized to include a consistent set of 16 cortical and subcortical brain regions with mRNA sequencing performed on 11,346 sets of carefully curated homologous genes (9). All but five of these genes were also present in the Allen Brain Atlas discovery dataset, and only the missing five were excluded for subsequent analysis. Similarly, to replicate developmental findings across species, we used a second pre-curated dataset, downloadable from the same link, labeled "Developmental rhesus and human data". This dataset, described in (9), includes tissue samples from 36 human brains (15 female, ages 8 post-conception weeks to 40 years, mean = 97 +/- 147 post-conception months) and 26 macaque brains (8 female, ages 60 post-conception days to 11 years, mean = 36 +/- 46 post-conception months). The dataset includes brain regions extracted from 16 cortical, subcortical and cerebellar brain regions across both species. Three transient developmental brain regions were excluded (lateral, medial and caudal ganglionic eminence), while other prenatal regions were considered equivalent to their most similar adult brain regions (e.g. dorsal thalamus to mediodorsal thalamus, upper rhombic lip to cerebellum, etc). This was only relevant for two of 62 total brains that possessed these early developmental regions. Each brain region had mRNA sequencing performed on 27,932 genes, of which 13,113 overlapped with the Allen Human Brain Atlas discovery set and were used for analysis.

Finally, to extend our investigation of conservation of molecular gradients beyond primates and into other mammals, we used a pre-curated(10) and preprocessed(11) postnatal mouse brain atlas dataset. This dataset included in situ hybridization of 2,069 genes from 434,946 brain sections of C57Bl/6J mice, which were registered to Allen Brain Atlas voxel space. Further detailed preprocessing information can be found in (11); data (12) and code (https://github.com/DynamicsAndNeuralSystems/DevelopingMouse) are freely available. K-nearest neighbors was used to impute missing values such that all voxels had data for all genes. Of the 2,069 available genes, there were 1,576 human homologs available in the original Allen Human Brain Atlas dataset, and this set was used for subsequent analysis.

PLS Methods

Model fitting, optimization and validation. Observations in developing brains suggest the presence of multiple large-scale directional gene expression gradients that exist

within and across compartments (**Fig 1A**), and which help to coordinate developmental programming and functional specialization. The current study investigated whether such gradients are present in the adult brain. Our objective was therefore to identify patterns of autocorrelated brain gene expression that vary systematically along a 3-dimensional linear axis. To accomplish this goal, we used a cross-decomposition framework in order to determine modes of latent covariance between gene expression (**X**; a 3466 sample by 15,634 gene matrix) and euclidean space (**Y**; a 3466 sample by 3 dimension matrix). Under a prediction framework, such a model would use latent gene expression components to predict the 3-dimensional location (i.e. x-,y- and z- coordinates) of a tissue sample in space (**Fig S1A**). However, the gene expression component (PLS X) in this case will be a latent variable representing linear variation in gene expression across a 3-dimensional axis – a construct consistent with the transcriptomic gradients we wish to characterize. Note here that, due to left-right symmetry relative to other brain dimensions, absolute x-coordinates were used in model fitting. This is equivalent to projecting all tissue samples onto one hemisphere, but otherwise maintaining their distance from the origin and y- and z-coordinate. True x-coordinates were used for display purposes only. This process should result in spatiomolecular gradients. In other words, as one moves in any of the three canonical directions outlined by the model, gene expression becomes more and more different. Importantly, this is not true for the whole transcriptome, but for a key set of genes. And the key set of genes that varies across distance is different for each gradient

As a first step, principal components analysis (PCA) was used to decompose the gene expression data into its first 100 components. These 100 components collectively explained 84.6% of the original data and reduced the input matrix X to a 3466 sample by 100 component matrix (**Fig S1A**). The choice to precede cross-decomposition with this data reduction step was made for the following reasons: i) recent benchmarking work suggests the stability of cross-decomposition weights is greatly reduced as a function of feature/sample ratio, such that a smaller feature/sample ratio is desirable (13); ii) gene expression data exhibits a hierarchical covariance structure with biologically meaningful co-expression networks at various scales (4); iii) computational efficiency is greatly increased, allowing for rapid permutation and ease of reproducibility.

Next, a 70/30 train-test split was applied to the dataset. The Allen Brain Atlas dataset provides 212 structural labels indicating the exact brain region the anatomist extracted the tissue from. The train/test split was stratified such that each of these tissue samples was equally distributed across the training and test sets. Across splits, we ensured components were inverted so as to match the direction of the full sample output. The following optimization procedures were executed exclusively on the n = 2,599 sample training set. A miniature grid search assessed model performance, estimated using both R^2 score and mean squared error, varying across different estimators (partial least squares regression [PLSR], partial least squares canonical

[PLSC] and canonical correlation analysis [CCA]) and number of components (1, 2, 3; note that 3 is the y matrix rank and therefore the maximum allowable number of components). For each of these nine parameter sets, out-of-sample performance was obtained using ten sets of ten-fold cross validation. This analysis revealed a three-component PLSR model to provide the best out-of-sample performance (**Fig S1C**), which was used for subsequent analysis. For completeness, this process was repeated without the initial PCA data reduction step, but this resulted in poorer model performance.

To ensure each component predicted unique variance greater than chance given the data structure, the 3-component PLSR was fit 1000 times on permuted data (i.e. with shuffled labels) to create a null model distribution (**Fig S1D**). Given the spatially autocorrelated nature of gene expression data, it is common to perform "spatially-aware" null models (14) that create null permutations with spatial autocorrelation consistent with the initial dataset. However, such an approach is inappropriate in this context given that autocorrelation is the target of the present analysis rather than a confounder to it. Instead, we validated the PLS LVs by reproducing them in several external datasets (see below).

We assessed the final model fit by using the 3-component PLSR model fit to the entire training set and using it to predict the x-, y- and z-coordinates of the n=867 sample left out test set. Once satisfied with the predictive performance of the model, we fit the model to the entire dataset and obtained predicted x-, y- and z- coordinates for all tissue samples. We also calculated the absolute error (predicted - actual) in mm of every sample along all three axes (x,y,z), as well as the mean of these three values. The mean error was correlated with a measure of extremity, representing the mean distance between a sample and every other sample. We also correlated error against distance from origin, measured as the distance of the sample to MNI coordinates [0,0,0].

We also performed a robustness test to ensure the PLS results were not somehow influenced by the intrinsic orientation of the brain (**Fig S1F**). In the training set, we performed 100 random rotations of the brain and applied these rotations to the y, z, and (absolute) x coordinates of each sample. We then conducted the same three-component PLS analysis between gene expression and Euclidean coordinates, though this time the Euclidean coordinates were randomly rotated. We compared empirical LVs rotated LVs by looking at correlations between their PLS X loadings. If the empirical (unrotated) spatiomoelcular gradients were robust, we would expect the latent Y (spatial) variables to change but the latent X (gene expression) variables to remain consistent. For each new rotated PLS model, we sequentially (from first to last rotated LV) assigned each LV to its most similar (remaining) empirical LV by finding the (remaining) empirical LV to which the rotated LV had the highest PLS X weight correlation. Once an empirical LV was matched, that LV was removed for the next LV in

the sequence. In other words, if rotated LV1 was most similar to empirical LV1, rotated LV2 was only compared to empirical LV2 and LV3. Once all rotated LVs were assigned, we created a correlation matrix of PLS X loadings of all 300 LVs (3 LVs x 100 rotations), and ordered them by their previously described empirical LV similarity. This showed that gene-expression LVs were highly similar across permutations despite spatial rotation. Furthermore, the rotated LVs were highly similar to the empirical LVs; the mean R^2 representing shared variance between empirical rotated and X loadings was 0.91 for LV1, 0.71 for LV2 and 0.78 for LV3 (**Fig S1F**).

Evaluation of PLS latent variables. Bootstrap resampling with replacement was used to generate confidence intervals around loadings for each of the three PLS latent variables (LVs). The confidence intervals were used to generate p-values representing the likelihood that the loading crosses 0, and these p-values were subsequently FDR corrected across loadings. To further characterize genes contributing to each component, loadings with FDR Q>0.05 were considered unstable and were set to 0. These newly regularized PLS component loadings were then transformed back into individual gene space by finding the dot product between the loading vector and the transposed principal component matrix, the latter of which was standardized before multiplication (**Fig S1B**).

To evaluate whether PLS LV expression varied meaningfully with aspects of brain developmental spatial organization, each tissue sample was categorized based on its anatomical label (**Dataset S7**). For visualization purposes, samples were given a broad spatial categorization into one of the following areas: Frontal, Temporal, Occipital, Parietal, Sensorimotor, Limbic, Subcortex, Brainstem, Cerebellum. In addition, samples were given a label based on their cortical type: association cortex, sensory cortex, cerebellar cortex, or not cortex. Samples were further categorized based on whether the region from which they were extracted originates from the myelencephalon, metencephalon, mesencephalon, diencephalon or telencephalon. Similarly, for all samples not categorized to the telencephalon, samples were categorized based on whether the region of extraction originates in the dorsal or ventral plate of the developing brain. Based on the hypothetical expression pattern of LV1 and LV2 (**Fig 1A**), LV1 expression was compared to the neural tube segment categorization, and LV2 to the dorsal/ventral plate categorization. Finally, the cortical expression of LV3 was compared to cortical T1w/T2w ratio (see section “**Comparison to canonical brain features”** below for details of surface representation of PLS LVs and T1w/T2w ratio).

Out-of-sample replication procedure. Several external datasets include similar gene sets to the Allen Human Brain Atlas, allowing for harmonization of the PLS X variables. In contrast, x-y-z coordinates were not available in external datasets, and spatial sampling was considerably sparser. However, given that the gradients defined in these

analyses span the entire brain, we expected that the spatial distribution of samples in external datasets was wide enough to detect the presence of the gradients.

The replication procedure (**Fig 1I**) involved the following steps: First, PLS-derived gradients were downsampled to match the resolution of external datasets by finding the mean value (for each component) of all samples falling inside of regions sampled in external datasets (see **Dataset S7** for mapping between Allen samples and regions from external dataset). For example, for replication in the BrainSpan dataset, mean values were derived from within the frontal, temporal, occipital, and parietal lobes, sensorimotor cortex, striatum, thalamus and cerebellum. Importantly, correlations were never performed across any analysis described below (group or individual) when n regions < 6.

Next, PLS X gradients were derived in external samples. This involved refitting both the PCA and the PLS model in the Allen Human Brain Atlas discovery set using only genes that overlapped between both the discovery and replication sets. 10-fold cross-validation was employed in the discovery set to compare overall performance of this PLS model compared to the original model. Next, the newly fit PC model was used to transform the replication samples to achieve a sample by (gene expression) component matrix, and the newly fit PLS model was then applied to this matrix. Note that prior to this step, the replication dataset was MinMax transformed so that all values fell between 0 and 1, using samples most similar to the Allen Human Brain Atlas dataset age range as the reference sample. Specifically, adult samples were used as the reference for BrainSpan, “younger” healthy controls (see above) were used as the reference for GTEx, and adult humans were used as reference in the PsychEncode datasets. This entire procedure together resulted in expression of each PLS latent variable for each sample in the replication dataset.

Finally, replication strength was evaluated by finding the correlation between regional PLS X expression in the (downsampled) AHBA data and PLS X expression in the external dataset. In effect, this analysis determines the degree to which the genes associated with a PLS latent variable are expressed in a regionally consistent manner across both datasets. A higher correlation indicates regional gradient expression more similar to the AHBA dataset. Consistent expression indicates a similar gradient exists in the external dataset (insofar as brain regions share the same spatial distribution), and further allows analyses to be run in the external dataset with the assumption that the gradient at hand is consistent with that described in the discovery dataset. To evaluate how well the gradients generalized across datasets, this replication procedure was performed specifically on the mean of all adult human samples in three separate datasets: BrainSpan, GTEx and PsychEncode (see **Replication cohorts**). Importantly, this procedure can be applied to test the consistency of a full dataset to the Allen dataset (by averaging expression across donors), but can also be applied at the individual donor level.

Cortical surface analysis and transcriptomic territories

Surface rendering. The PLS analysis was conducted on samples extracted from the entire brain, including cortex, subcortex, midbrain, cerebellum and brainstem. However, we were interested in assessing whether whole-brain gradients converge in any functionally meaningful way in the cerebral cortex, where transcriptomic variation is considerably less pronounced (1). For each PLS X component, a 7 mm cube was created centered around the MNI coordinates of each tissue sample, where the values corresponded to PLS X expression of that component. This set of cubes was saved as a nifti file and converted to a fslr32k left hemisphere surface using connectome workbench's "volume-to-surface-mapping" command. Values were then interpolated across the cortical surface using the "metric-smoothing" with the "fix-zeros" flag and a 5 mm kernel, so that all vertices contained values for each gradient.

Derivation of canonical brain features. We were interested in whether gradient distribution could explain the topography of other canonical cortical features. Canonical features chosen were those used in Sydnor et al. (15): T1w/T2w ratio, evolutionary expansion, allometric scaling, cerebral bloodflow, Neurosynth PC1, externopyramidization, aerobic glycolysis, cortical thickness and Allen Human Brain Atlas PC1. To this list of features, we added maps for average geometric distance as described in Margulies et al. (16), developmental cortical expansion, magnetoencephalography intrinsic timescale, and gene expression-associated cognitive functional activation as described in Hansen et al. (17). **Dataset S3** describes each feature and the paper and dataset from which they are sourced. Values for each feature were available using the Glasser parcellation (18).

Comparison to principal fMRI gradients. Recent work suggests the principal functional gradients, derived using data-driven decomposition of resting-state functional connectivity (16, 19), represent a fundamental cortical hierarchy that discriminates several functional, morphological and biological features of the brain (15). Given that the gradient territories derived in this analysis also discriminated brain features, we conducted a direct comparison between these organizational brain maps in predicting the distribution of different cortical features. To achieve this goal, for each of the brain features previously discussed, we fit three ordinary least squares linear models with brain features as dependent variables; one used the three molecular gradients from the present analysis as independent variables, one used the first three principal functional gradients (16), and one used all six of these maps. We recorded the total explained variance (R2) of each model. To test whether one model explained significantly more variance than another, we performed bootstrapping resampling (100 iterations with

replacement) to generate confidence intervals. A significant difference was recorded when the lower 95% CI of the higher distribution did not overlap with the mean of the lower distribution.

Clustering analysis and molecular territories. Clustering analysis was used to find empirical and data-driven territories of gradient overlap (**Fig S7A**). The cortical array of each PLS X component was concatenated creating a 32,492 vertex x 3 component matrix, which was normalized with a MinMax scaler so that all values fall between 0 and 1. This matrix was subjected to hierarchical agglomerative clustering using euclidean distance and ward criterion for linkage, varying number of clusters (k) between 2 and 15 clusters. Silhouette score (20) and Calinski-Harabasz score (21) were used to evaluate the fit of each clustering solution, where "peak" solutions with better scores relative to surrounding solutions were sought. Both score types converged in demonstrating a peak at k=6 (**Fig S7A**). Labels from the 6-cluster solution were subsequently obtained for each vertex and visualized. For the purposes of interpretation, the distribution of each PLS X component was visualized for each cluster. To compare the molecular territories to canonical cortical lobes, a glasser-space parcellation was obtained that assigned each parcel to either the parietal, occipital, frontal or temporal lobe (22). This map of lobar labels was compared directly to the glasser-space parcellation of molecular territory labels using adjusted mutual information score and adjusted Rand index – two approaches to compare two clustering solutions. To compare these results to chance, we calculated the same scores for 1000 spatially-aware (i.e. spun) null permutations (see section **Comparison to electrophysiological brain activity** below) of the molecular territory map.

To see whether whole-brain information was truly contributing to cortical territories above and beyond cortical information, we repeated several of the aforementioned analyses, this time restricting data to samples extracted from the cerebral cortex (i.e. excluding samples from subcortex, cerebellum and brainstem). PLS was rerun using only cortical samples, and three LVs were extracted and projected to the cortical surface. Spatial correlations were used to determine the similarity of LVs generated using just cortical samples to those generated using all samples. The same hierarchical clustering pipeline as described above was once again used to generate cortical territories based on cortical-only LVs. These new cortical-only cortical territories were also compared to the canonical lobes using the Rand index and adjusted mutual information. Finally, using the same methods described in the above section "**Comparison to principal fMRI gradients"**, we directly compared cortical-only LVs to whole-brain LVs in explaining the spatial distribution of various cortical features. Models explaining significantly more variance in individual features using whole-brain vs. cortical only LVs (assessed using bootstrap tests) are reported.

Neurosynth decoding. We performed neurosynth decoding to further establish relevance of transcriptomic territories to functional organization. The objective of this analysis was to ascertain behavior-relevant terms associated with meta-analytic functional activation of regions falling within each transcriptomic territory. The neurosynth v4-topics-100 association maps were downloaded from (https://neurosynth.org/analyses/topics/v4-topics-100/). Each of these 100 maps represents regional meta-analytic functional coactivation associated with a set of associated terms, derived using latent dirichlet analysis (see (23) for details). Thirty-two maps were removed from the analysis because the associated terms were not associated with behavior (see **Dataset S8**), Each map was subsequently converted from volume to surface using the same approach as above (but without the interpolation step). For each topic map, the mean value was extracted within each transcriptomic territory. The top 5 topic maps for each territory were recorded and visualized.

Comparison to electrophysiological brain activity. Seven magnetoencephalography (MEG) cortical surface maps were downloaded using the Neuromaps software (24). These maps approximate local field potentials from cell populations by detecting electromagnetic cortical activity occurring at multiple wavelengths. This data was measured from 100 individuals as part of the Human Connectome Project (25), and was processed using the Brainstorm software (26) as previously described (27). Maps were available for the following canonical frequency bands: alpha (8–12 Hz), beta (15-29 Hz), delta (2-4 Hz), theta (5–7 Hz), low gamma (30-59 Hz), high gamma (60-90 Hz), as well as intrinsic timescale (24, 28). These maps were resampled from Freesurfer 4k space to fslr 32k space using nearest neighbor interpolation, and were parcellated using the Glasser atlas. Regional correlations were conducted between each MEG map and each of the three molecular gradients. The highest correlation for each gradient was visualized and significance was tested using a spatially-aware permutation test. Specifically, an established permutation framework to account for spatial autocorrelation (29, 30) (https://github.com/frantisekvasa/rotate_parcellation) was applied to each of the three molecular gradients to create null brain maps. Each map was correlated to the MEG map with the highest correlation to that gradient, creating a null distribution of r-values. The observed statistic using the true gradient map was compared to this null distribution to derive exact p-values. All correlations between molecular components, MEG maps, and fMRI gradients can be found in **Fig S8**.

Expression of gradients across development and across species

Cross-species gradient expression. We were interested in the degree to which the molecular gradients defined in the Allen would replicate in non-human primate adults, indicating to what degree the gradients are specific to humans. Using the

PsychENCODE Human Brain Evolution Adult human, chimpanzee, macaque dataset (see **Replication cohorts**), we calculated correlation values (see **Out-of-sample replication procedure**) for each component across six human, five chimpanzee and five macaque donors, all adults. This resulted in *r*-values for each individual that, for each component, represented the degree to which the individual regional component expression correlated with that of the discovery dataset. We used linear models to evaluate whether there were main effects of species and PLS latent variables, as well as their interaction. To ensure correlations were not a product of the data distribution and low number of tissue samples (n[max]=16), we create individual-specific null effect distribution by, for each individual, permuting their data without replacement and re-running the correlation 100 times. These null distributions were used to define analysis-specific p-values for each individual indicating the likelihood the observed inter-dataset correlation value was achieved by chance given a random set of genes.

We also wanted to ensure that high reproducibility of the gradients was not simply driven by dataset similarity. In other words, we were curious whether the gradient replication is consistent with replication of any coherent biological signal derived from the Allen dataset. To assess this, we created a distribution of biological signals by finding the first 100 principal components in the Allen Human Brain Atlas dataset. Using the same procedure described above (**Replication procedure**), we found correlation values for each individual across all 100 components, and used these values to create individual-specific biological signal distributions. We used these distributions to derive "p-values" for each PLS gradient representing the likelihood that individual-level reproducibility of the gradient exceeds that of the average biological signal (in this case, the average Allen Human Brain Atlas principal component).

Finally, we performed an analysis to explore conservation of molecular gradients in mice. The PLS model (X gene expression, Y 3D coordinates) was fitted on the discovery human dataset once again, this time using only the 1,576 genes shared (through homology) between the mouse and human datasets. The fitted model was applied to the mouse gene expression data to predict coordinates of each voxel. Predicted and observed y, z and x coordinates were compared. Next, the model fitted to the human data was used to transform the mouse gene expression data (see **Out-of-sample replication procedure** above**)**, effectively resulting in voxelwise projections of each human LV onto the mouse brains. The distribution of the LVs were qualitatively compared to the distribution seen in humans. To probe whether the conservation of gradients across species was driven by conserved gradient-related genes, we ran spatial correlations between each gene in the mouse brain atlas and each of the three projected LV patterns. For each LV, we selected the top and bottom 0.8% of correlations (see section **Gene list curation** below), and we cross-referenced these top LV-associated genes with the top LV-associated genes of the same LV in humans. For

each component, we report genes that appear on both the human and mouse top LV-associated gene list.

Developmental gradient expression. We were interested in tracking the emergence of each gradient during brain development, and determining approximately at what developmental epoch these gradients demonstrated adult-like regional expression. In the Brainspan dataset (see **Replication cohorts**), we once again derived correlations for each individual representing similarity to the discovery dataset. In this case, since the discovery dataset was composed of only adult brains, this correlation value can be thought to represent the degree to which gradient expression in the individual resembles average adult gradient expression. These donor-level correlation values were then plotted against log age. To remove 0s, before log transformation, age in both GTEx and Brainspan age was calculated in weeks with week 0 equal to conception (assuming a 40 week pregnancy). To ensure results were interpretable with respect to true age, samples were divided into age groups: fetal (prenatal), infant (0-2 years), child (2-10 years), adolescent (11-19 years) or adult (20+ years). To ensure the observed developmental trajectories were generalizable across datasets, and to evaluate whether they were generalizable across primate species, we repeated the procedure described above for individual human and macaque brains from the PsychENCODE "Developmental rhesus and human dataset" (see **Replication cohorts**). For macaques, weeks start at conception assuming a 24 week gestation period. Age groups for macaques were constructed as follows: fetal (prenatal), infant (0-1 years), child (2-3.5 years), adolescent (3.5-7 years), adult (8+ years).

The molecular gradients investigated presently were defined in the adult brain, though it is likely that many of the genes contributing to the establishment and regulation of these gradients are expressed only during certain phases of neurodevelopment. Ideally, a PLS model similar to the one used in the adult Allen Brain dataset could be used to determine independent LVs in each of the other developmental epochs, but spatial sampling in the prenatal datasets was not nearly dense enough to allow such an investigation. Instead, we grouped samples into three categories (prenatal, postnatal, adult) and we searched for genes that were expressed in a pattern resembling the adult LVs within these developmental epochs. Using the same approach as above, for each epoch, we correlated regional expression of each gene with each of the three adult LVs from the discovery (Allen) dataset. As above, we found the top 0.8% positive and negative correlations (respectively) for each LV, took the mean of their absolute values within-donor, and plotted these values over neurodevelopment (i.e. against log age of the donor). This approach helped to visualize whether gradient-like gene expression is restricted to specific epochs or whether it is constant throughout development.

Identifying candidate early developmental regulators of spatiomolecular gradients. Despite discovering the spatiomolecular gradients using data from adult human brains, we hypothesize that these signatures reflect changes instantiated during early development. Therefore, we used a data-driven approach to identify and validate genes that show regional patterns resembling those of the spatiomolecular gradients already during early brain development (**Fig S11B**). We hypothesized that a proportion of gradient-associated genes would show regional expression patterns resembling the LVs throughout development, including during prenatal periods. Using the Brainspan data as the discovery dataset, we repeated analyses described in Methods section "**Developmental gradient expression"** to derive regional similarity to adult (AHBA) gradients, but for each LV-associated gene across each donor. This process resulted in a developmental trajectory of gradient similarity for each LV-associated gene. For each LV, these data were then clustered using agglomerative clustering with ward criterion (**Fig S11B**). Clustering was repeated for k = 2 to 50, and silhouette index was derived from each clustering solution. Peaks in silhouette index across solutions were identified and examined visually by averaging genes within the same cluster and plotting their expression across age (**Fig S11B**). The lowest k solutions to demonstrate a cluster with high regional similarity to its LV throughout the measurement period (e.g. a non-transitional developmental pattern (31)) was selected (**Fig S11B**). A k=12 solution was selected for LV1, a k=7 solution for LV2, and a k=17 solution for LV3.

Next, a technique was implemented to "apply" the clustering solution derived in brainspan to the PsychENCODE human data. For selected clustering solutions, cluster centroids were derived for each cluster. This was accomplished by plotting the mean expression of genes within the cluster against log age, and then deriving the fitted lowess curve summarizing this relationship. Next, the fitted lowess curve of expression vs age was derived for each gradient-associated gene in the PsychENCODE dataset. For each gene, mean absolute distance was calculated between its fitted curve and that of each cluster centroid. The gene was then assigned to the cluster for which the shortest distance was observed between the gene's curve and centroid curve. After this process was repeated for all genes, genes were identified that fell into the non-transitional cluster in both datasets (**Fig S11B**). This set of genes was further curated by eliminating any gene that showed a negative regional correlation with adult LV expression across any samples, and eliminating any gene that never showed a regional correlation with adult LV above 0.5 across any sample. This process resulted in eight genes for LV1, 11 genes for LV2 and 1 gene for LV3 that showed high regional similarity to adult LV expression across brain development.

Finally, we wished to assess whether genes identified in the previous analysis also showed consistent adult LV-like expression throughout development in macaques. This required us to map macaque developmental time to human developmental time, which was accomplished using harmonized developmental periods (9, 32). We then

visualized regional similarity to LV expression across developmental periods for each of the 20 genes identified from the previous analysis across all three datasets (Brainspan, PsychENCODE Macaque, PsychENCODE Human).

Annotation of gradient-associated genes

Gene list curation. Genes were identified as significant contributors to each gradient. Significant contribution was defined as the top and bottom 0.83% genes (n=260) of each gradient's gene list, sorted by PLS X loading. This represents the 5% most contributing genes, divided in half (as both tails were assessed), then divided by three (for multiple comparisons, as three components were assessed). This produced six different gene sets, but for most analyses, top genes were combined across all three components. This gene set is referred to throughout the manuscript as "gradient-associated genes". Each gene was further categorized based on whether it was part of only one component's (both tails) gene sets, was present in two components gene sets, or was present in all three.

Gene enrichment analysis. Two gene ontology enrichment analyses were conducted. The first evaluated the enrichment of a combination of all 780 top gradient-associated genes. The second analyses involved separate analyses of three different gene sets defined by whether genes contributed to one, two or all three components (see above). For each analysis (four in total), gene lists were submitted as gene sets to ToppGene's ToppFun enrichment feature (https://toppgene.cchmc.org/enrichment.jsp), using the whole list of Allen Human Brain Atlas genes as the background gene set. Only the following term categories were assessed: GO: Molecular Function, Go: Biological Process, Go: Cellular Component, Pathway (all), Disease (all), and Human Phenotype. All other settings were left to their defaults. Note that ToppGene databases are continuously updated; this ToppGene query was conducted on June 13, 2022. The gene set enrichment analysis further nominated associations with several psychiatric diseases. To validate this finding, we used an established gene-set analysis method (MAGMA) (33) to evaluate the enrichment of gradient-associated genes against a published genome-wide association study identifying two distinct factors across multiple psychiatric conditions (34).

Post-hoc morphogen analysis. The gene set enrichment analysis revealed several terms relating to brain development. We also had an a priori hypothesis that the gradients should include developmental morphogens (see **Introduction**). Therefore, we identified a recently published list of genes expressed in a gradient-like pattern in the ventricular zone of the developing murine brain (35). We counted the proportion of these genes that overlapped with gradient-associated genes from our study. A p-value

for enrichment was calculated by comparing this overlap with that of 1000 permuted gene sets (without replacement) of the same length as the gradient-associated gene set (n=780).

mmQTL-based PheWAS. We wished to further explore the relationship between gradient-related genes and aspects of function and behavior. Cross-referencing of gradient-associated genes was performed against a database of multi-ancestry meta-analytic quantitative trait loci (mmQTL), which had systematically been linked to phenotypes via phenome-wide association studies (PheWAS) (36). Associated files were downloaded from Synapse (https://www.synapse.org/#!Synapse:syn23204884/wiki/606411). This data represented SNPs that were i) associated with both brain expression of a gradient-related gene and ii) genome-wide significantly related to a trait. We identified gradient-related genes from this data resource and created a Sankey diagram to summarize two-way associations.

Data and code availability.

All analyses in this manuscript were performed using pre-existing datasets, and each of which can be accessed online (see Methods). All figures, tables and analyses can be reproduced using a set of Jupyter notebooks and supplemental scripts: https://github.com/PennLINC/Vogel_PLS_Tx-Space.

SI Results

Further characterization of the three latent variables. The first component (LV1) featured gene expression varying along a rostro-caudal axis, radiating from the brainstem and cerebellum, through the midbrain, subcortex and posterior cortex, and finally into anteriodorsal cerebral cortex. Expression of this component demonstrated linear rostro-caudal increase as one moves along regions originating from different segments of the neural tube (**Fig S4C**). The second component (LV2) was expressed in a whole-brain dorsal-ventral pattern, with highest expression in the brainstem, subcortex and frontal cortex, and lowest expression in cerebellar and posterior cerebral cortex. Within regions originating from neural tube segments, LV2 expression differed between regions originating from the dorsal and ventral plates (**Fig S4D**). The third component (LV3) described gene expression varying along a gradient diffusing in a medial-lateral and dorsal-ventral direction. Of the three gradients, the LV3 gradient showed the most variation within the cerebral cortex (**Fig S4A**). The cortical expression of the LV3 gradient also bore a strong relationship to the pattern of cortical myelination, which has been shown to be a robust index of hierarchical functional organization (37, 38) ($r=0.78$, $p<0.001$; **Fig S4E**).

Interestingly, each PLS latent variable had a unique distribution of cortical expression (**Fig S4F**). Low LV1 expression differentiated the cerebral cortex from the rest of the brain. Meanwhile, LV2 expression was high in non-cortical structures, strongly differentiating it from cortex (notably including cerebellar cortex). Finally, LV3 expression was highest particularly in different types of sensory cortex, differentiating it from other parts of the brain, including association cortex. These analyses demonstrate how association cortex, sensory cortex, cerebellar cortex, and non-cortical structures show distinct patterns of spatiomolecular organization in the human brain.

Conservation of gradients across species not a product of general transcriptomic conservation. We tested whether the high similarity in spatiomolecular axes observed across human, chimpanzee and macaque brains was simply a product of generally conserved brain gene expression patterns across the three species, or whether conserved expression was especially enhanced for our three spatiomolecular axes. In other words, we wished to deduce how reproducible or conserved these gradients are compared to other spatial components of biologically covarying genes. Therefore, we derived 100 components of brain regional gene coexpression in the discovery dataset using principal components analysis. We used the interspecies reproducibility of these 100 components as a distribution against which to compare the expression pattern of the three spatiomolecular gradients, and we performed this analysis separately across each individual human, macaque and chimpanzee individual (**Fig S9B)**. Across all

individuals, we found that reproducibility of LV1 and LV2 to be significantly ($p<0.05$) greater than expected for gene coexpression networks across all individuals. Across both species, LV1 and LV2 expression were consistently in the top 5% of biological components in terms of reproducibility. LV3 was generally less reproducible, though reproducibility was still within the top 10% of biological components for all except one chimpanzee individual.

SI References

1. M. J. Hawrylycz, *et al.*, An anatomically comprehensive atlas of the adult human brain transcriptome. *Nature* **489**, 391–399 (2012).
2. A. Arnatkeviciute, B. D. Fulcher, A. Fornito, A practical guide to linking brain-wide gene expression and neuroimaging data. *Neuroimage* **189**, 353–367 (2019).
3. R. D. Markello, *et al.*, Standardizing workflows in imaging transcriptomics with the abagen toolbox. *eLife* **10** (2021).
4. M. Hawrylycz, *et al.*, Canonical genetic signatures of the adult human brain. *Nat. Neurosci.* **18**, 1832–1844 (2015).
5. B. D. Fulcher, M. A. Little, N. S. Jones, Highly comparative time-series analysis: the empirical structure of time series and their methods. *Journal of The Royal Society Interface* **10**, 20130048 (2013).
6. J. A. Miller, *et al.*, Transcriptional landscape of the prenatal human brain. *Nature* **508**, 199–206 (2014).
7. GTEx Consortium, The Genotype-Tissue Expression (GTEx) project. *Nat. Genet.* **45**, 580–585 (2013).
8. C. L. Hartl, *et al.*, Coexpression network architecture reveals the brain-wide and multiregional basis of disease susceptibility. *Nat. Neurosci.* **24**, 1313–1323 (2021).
9. Y. Zhu, *et al.*, Spatiotemporal transcriptomic divergence across human and macaque brain development. *Science* **362** (2018).
10. C. L. Thompson, *et al.*, A high-resolution spatiotemporal atlas of gene expression of the developing mouse brain. *Neuron* **83**, 309–323 (2014).
11. H. Y. G. Lau, A. Fornito, B. D. Fulcher, Scaling of gene transcriptional gradients with brain size across mouse development. *Neuroimage* **224**, 117395 (2021).
12. B. Fulcher, H. Y. G. Lau, Data files to support “Scaling of gene transcriptional gradients with brain size across mouse development” (2021) https:/doi.org/10.5281/ZENODO.4620136.
13. M. Helmer, *et al.*, On stability of Canonical Correlation Analysis and Partial Least Squares with application to brain-behavior associations. *bioRxiv*, 2020.08.25.265546 (2021).
14. R. D. Markello, B. Misic, Comparing spatial null models for brain maps https:/doi.org/10.1101/2020.08.13.249797.
15. V. J. Sydnor, *et al.*, Neurodevelopment of the association cortices: Patterns, mechanisms, and implications for psychopathology. *Neuron* **109**, 2820–2846 (2021).
16. D. S. Margulies, *et al.*, Situating the default-mode network along a principal gradient of macroscale cortical organization. *Proc. Natl. Acad. Sci. U. S. A.* **113**, 12574–12579 (2016).
17. J. Y. Hansen, *et al.*, Mapping gene transcription and neurocognition across human neocortex. *Nat Hum Behav* **5**, 1240–1250 (2021).
18. M. F. Glasser, *et al.*, A multi-modal parcellation of human cerebral cortex. *Nature*

536, 171–178 (2016).
19. R. Vos de Wael, *et al.*, BrainSpace: a toolbox for the analysis of macroscale gradients in neuroimaging and connectomics datasets. *Commun Biol* **3**, 103 (2020).
20. P. J. Rousseeuw, Silhouettes: A graphical aid to the interpretation and validation of cluster analysis. *Journal of Computational and Applied Mathematics* **20**, 53–65 (1987).
21. T. Calinski, J. Harabasz, A dendrite method for cluster analysis. *Communications in Statistics - Theory and Methods* **3**, 1–27 (1974).
22. A. S. Keller, A. V. Jagadeesh, L. Bugatus, L. M. Williams, K. Grill-Spector, Attention enhances category representations across the brain with strengthened residual correlations to ventral temporal cortex. *Neuroimage* **249**, 118900 (2022).
23. T. Yarkoni, R. A. Poldrack, T. E. Nichols, D. C. Van Essen, T. D. Wager, Large-scale automated synthesis of human functional neuroimaging data. *Nat. Methods* **8**, 665–670 (2011).
24. R. D. Markello, *et al.*, neuromaps: structural and functional interpretation of brain maps. *bioRxiv*, 2022.01.06.475081 (2022).
25. D. C. Van Essen, *et al.*, The WU-Minn Human Connectome Project: An overview. *NeuroImage* **80**, 62–79 (2013).
26. S. Baillet, K. Friston, R. Oostenveld, Academic software applications for electromagnetic brain mapping using MEG and EEG. *Comput. Intell. Neurosci.* **2011**, 972050 (2011).
27. G. Shafiei, S. Baillet, B. Misic, Human electromagnetic and haemodynamic networks systematically converge in unimodal cortex and diverge in transmodal cortex. *bioRxiv* (2021) https:/doi.org/10.1101/2021.09.07.458941.
28. R. Gao, R. L. van den Brink, T. Pfeffer, B. Voytek, Neuronal timescales are functionally dynamic and shaped by cortical microarchitecture. *Elife* **9** (2020).
29. F. Váša, *et al.*, Adolescent Tuning of Association Cortex in Human Structural Brain Networks. *Cereb. Cortex* **28**, 281–294 (2018).
30. J. Seidlitz, *et al.*, Morphometric Similarity Networks Detect Microscale Cortical Organization and Predict Inter-Individual Cognitive Variation. *Neuron* **97**, 231–247.e7 (2018).
31. M. Li, *et al.*, Integrative functional genomic analysis of human brain development and neuropsychiatric risks. *Science* **362** (2018).
32. H. J. Kang, *et al.*, Spatio-temporal transcriptome of the human brain. *Nature* **478**, 483–489 (2011).
33. C. A. de Leeuw, J. M. Mooij, T. Heskes, D. Posthuma, MAGMA: generalized gene-set analysis of GWAS data. *PLoS Comput. Biol.* **11**, e1004219 (2015).
34. T. T. Mallard, *et al.*, Multivariate GWAS of psychiatric disorders and their cardinal symptoms reveal two dimensions of cross-cutting genetic liabilities. *Cell Genom* **2** (2022).
35. A. R. Ypsilanti, *et al.*, Transcriptional network orchestrating regional patterning of cortical progenitors. *Proc. Natl. Acad. Sci. U. S. A.* **118** (2021).
36. B. Zeng, *et al.*, Multi-ancestry eQTL meta-analysis of human brain identifies candidate causal variants for brain-related traits. *Nat. Genet.* **54**, 161–169 (2022).
37. J. B. Burt, *et al.*, Hierarchy of transcriptomic specialization across human cortex captured by structural neuroimaging topography. *Nat. Neurosci.* **21**, 1251–1259 (2018).
38. N. A. Foit, et al., A Whole-Brain 3D Myeloarchitectonic Atlas: Mapping the Vogt-Vogt Legacy to the Cortical Surface. bioRxiv, 2022.01.17.476369 (2022).

Supplementary Figures

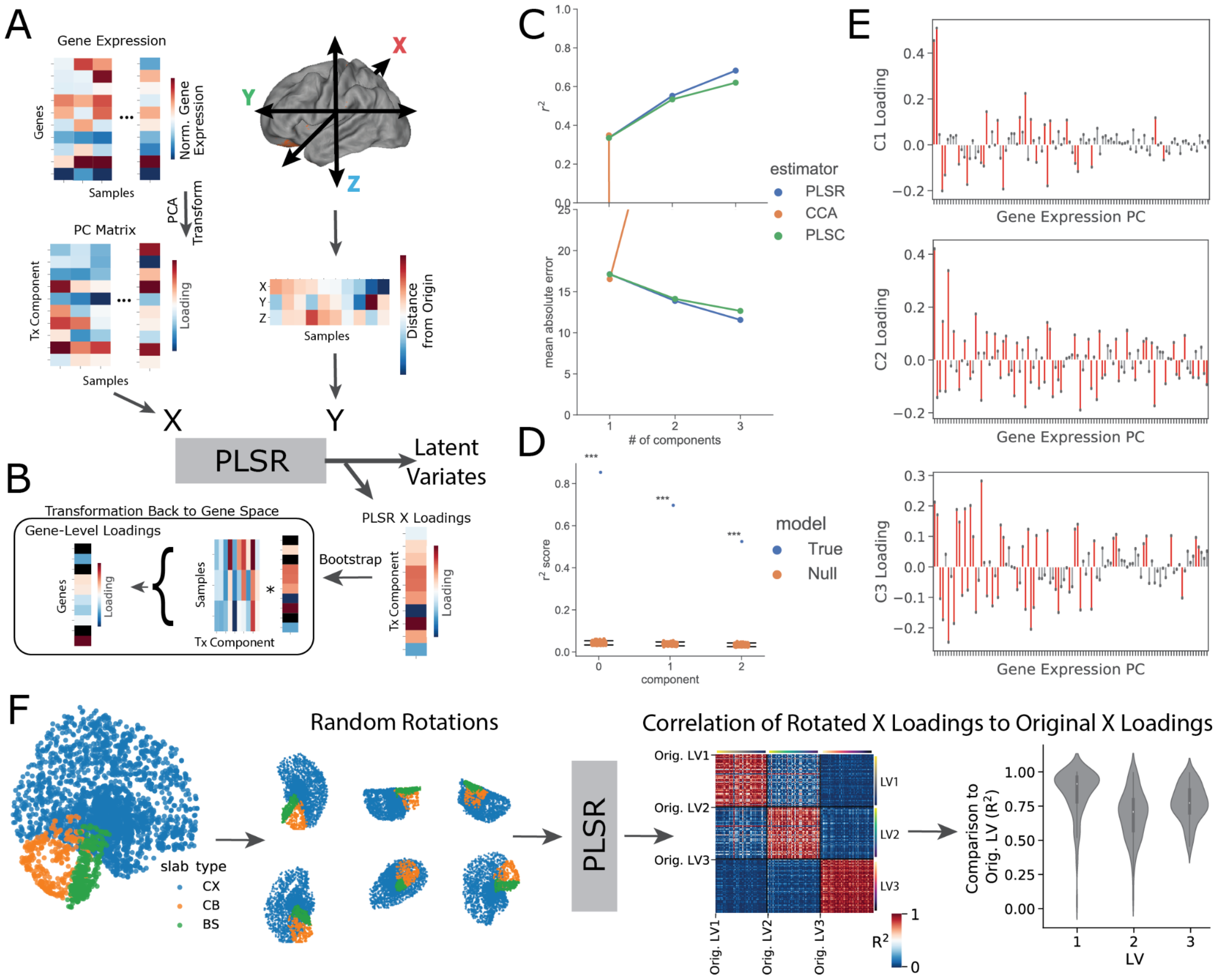

Fig S1. **PLS methods schematic. A)** PLS model was fit to multivariate relationships between gene expression and space. One side of the PLS (PLS X), a gene-by-sample matrix, was decomposed into 100 principal components. The other side (PLS Y) contained x (medial-lateral), y (anterior-posterior) and z (dorsal-ventral) MNI stereotaxic coordinates where x coordinates were converted to absolute values. The resulting PLS components are made up of latent linear combinations of gene expression (PLS X) and spatial positioning (PLS Y) that demonstrate maximal covariance (as in **Fig 1**). **B)** The dot product of the PLS rotation vector and the transposed standardized principal component matrix from A) was found, resulting in PLS X loadings in gene space. **C)** In the training set, 100 rounds of 10-fold cross-validation were used to optimize performance over three different estimators (PLS-R, PLS-C, CCA) and over number of PLS components (matrix rank for PLS Y in this case is 3, so C=1-3 evaluated). Point plots show R^2 (top) and MSE (bottom) across estimators (hue) and number of components (x-axis). Confidence intervals are present but not visible in the plot. CCA performed poorly when number of components > 1, whereas PLSC and PLSR showed improved performance with

increasing components. Top performance was achieved using PLSR with 3 components. **D)** The optimal model from C was rerun 1000 times with shuffled labels and explained variance of each component was recorded. This null distribution (orange dots) was used to determine if the explained variance of the empirical PLS model (blue dot) was greater than what would be expected by chance given the dataset. **E)** PLS X loadings for all 100 input features (gene expression principal components) for each of the 3 PLS latent variables summarized over 1000 bootstrap samples. Red-colored features indicate that the 95% CI of the mean feature weight across bootstrap samples did not cross 0, indicating good reliability. Importantly, PC1 (the first feature) did not dominate any PLS LV and was distributed fairly evenly across all 3 PLS LVs, indicating none of the PLS LVs are simply equivalent to PC1. **F)** To ensure molecular gradients were not driven by arbitrary spatial coordinates, (left) the brain was rotated 100 times and, for each rotation, the same gene expression by (rotated) x,y,z coordinate PLS was run. (right) The loadings of each set of X (gene expression) LVs were correlated. The correlation matrix shows the correlation (R2) between the X loadings of all three LVs for each rotation, as well as for the original (orig.; i.e. non-rotated) PLS model. The matrix is ordered first by LV, then by rotation, and clearly shows high within-LV and low between-LV correlations across rotations. The violin plot shows that the X loadings across rotations were highly consistent with those of the original PLS model. This indicates that spatiomolecular gene-expression gradients were not driven by base brain orientation.

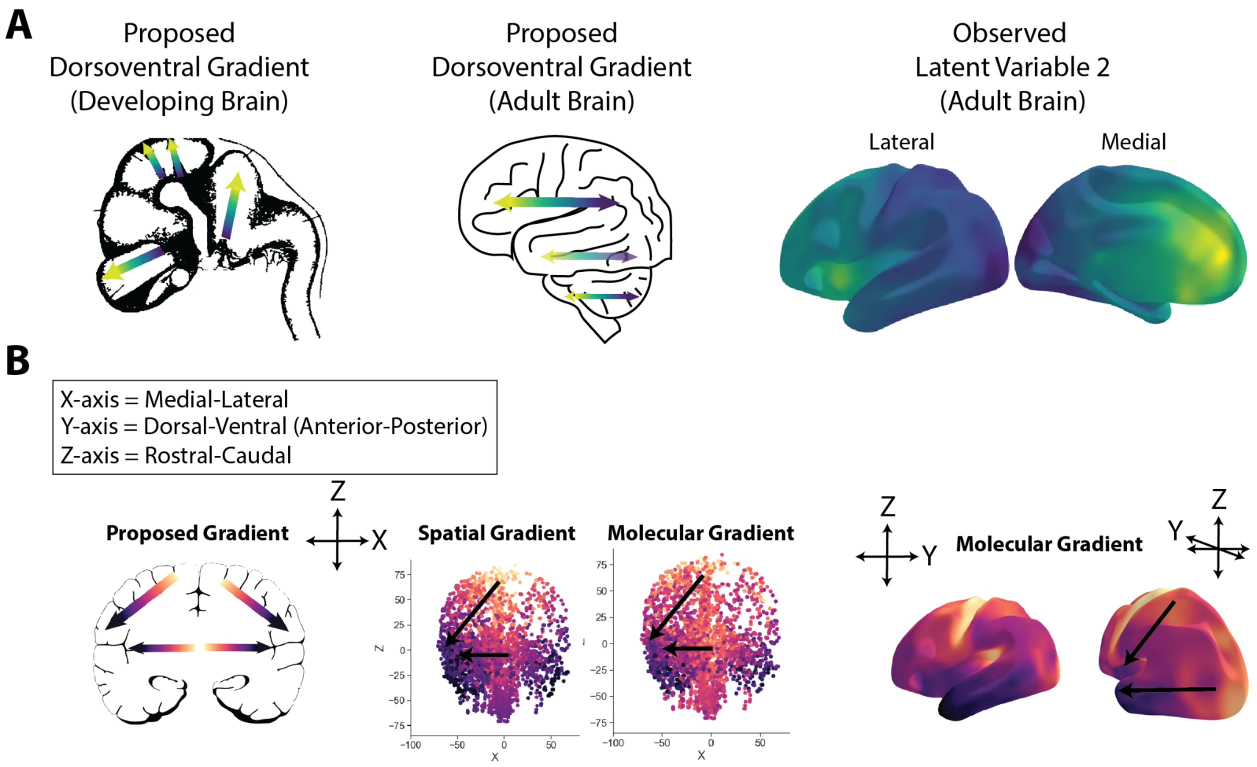

Fig S2. Further details on distribution of gradients. A) Hypothetical dorsoventral gradient in the developing (left) and adult brain (center), followed by cortical projection of observed LV2 in the adult brain (right). LV2 resembles the dorsoventral gradient (evident from the lateral view). Note also how the dorsoventral gradient in the developing brain manifests as an anterior-posterior gradient on the cerebral cortex. **B)** In the whole-brain projections from Figure 1, a coronal view is displayed where the medial-lateral (X) axis is visible. Here, arrows have been added to these views corresponding to the proposed directional gradients. On the right, a cortical rendering of LV3 from is displayed from a sagittal view. A 45-degree rotation is performed along the X-axis, so that the frontal lobe moves “away” while the occipital lobe moves “toward” the viewer. This makes the medial-lateral view visible (at the expense of the y-axis). From this view, the medial-lateral gradation of LV3 can once again be clearly appreciated. Arrows corresponding to the proposed gradients have once again been added to further aid with orientation.

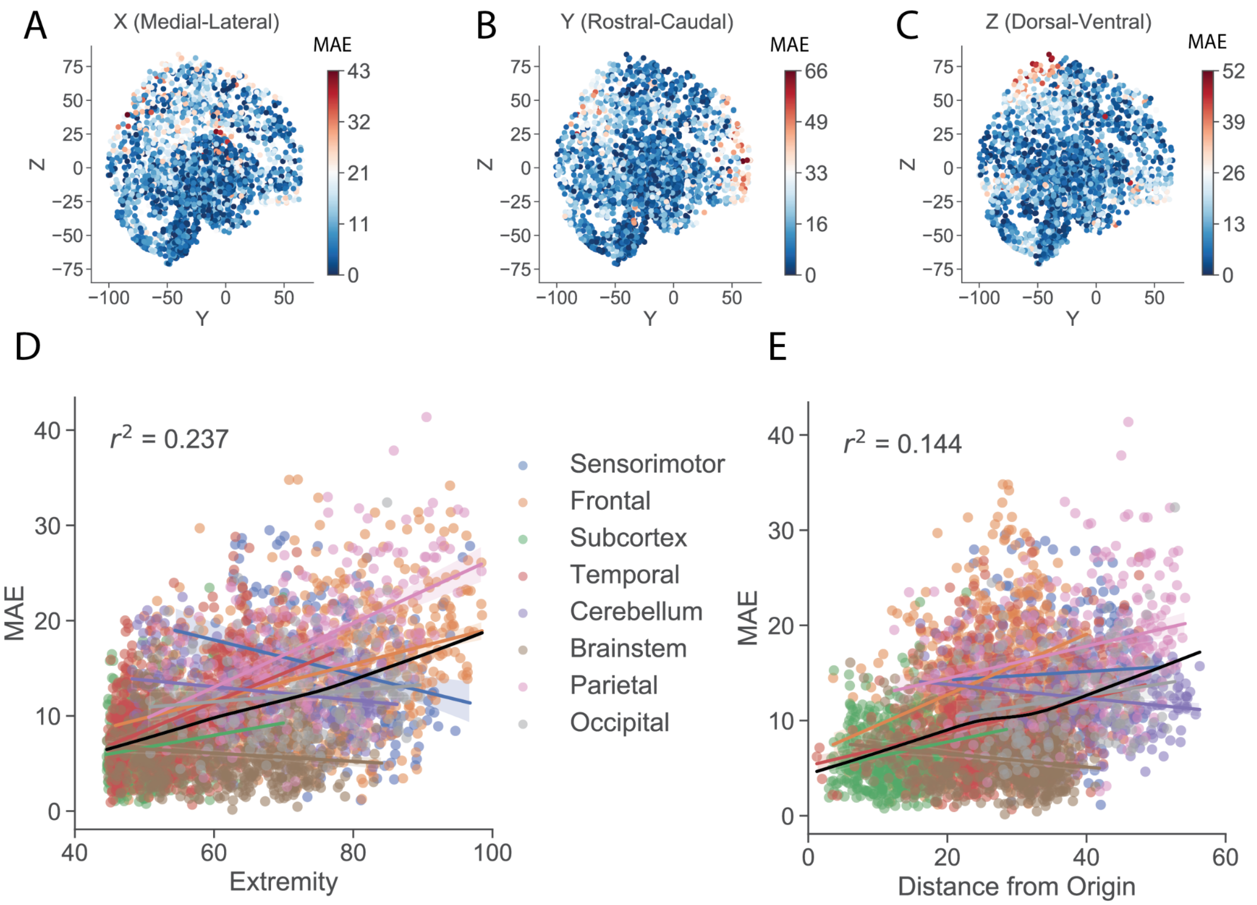

Fig S3. **Quantifying error in PLS model.** Figure 1 shows the overall absolute error associated with each sample. Here, error specifically within **A)** x-, **B)** y- and **C)** z-coordinates are visualized. **D)** Extremity of each sample was quantified as the average distance from all other samples. Extremity explained approximately 24% of the variance in sample error, such that the location of samples extracted from more extreme spatial locations of the brain were less accurately predicted. This relationship was driven especially by samples located in frontal, parietal and temporal neocortex. **E)** Similar to D), the location of samples located further from the origin (MNI coordinate 0,0,0) were more poorly predicted by the PLS model.

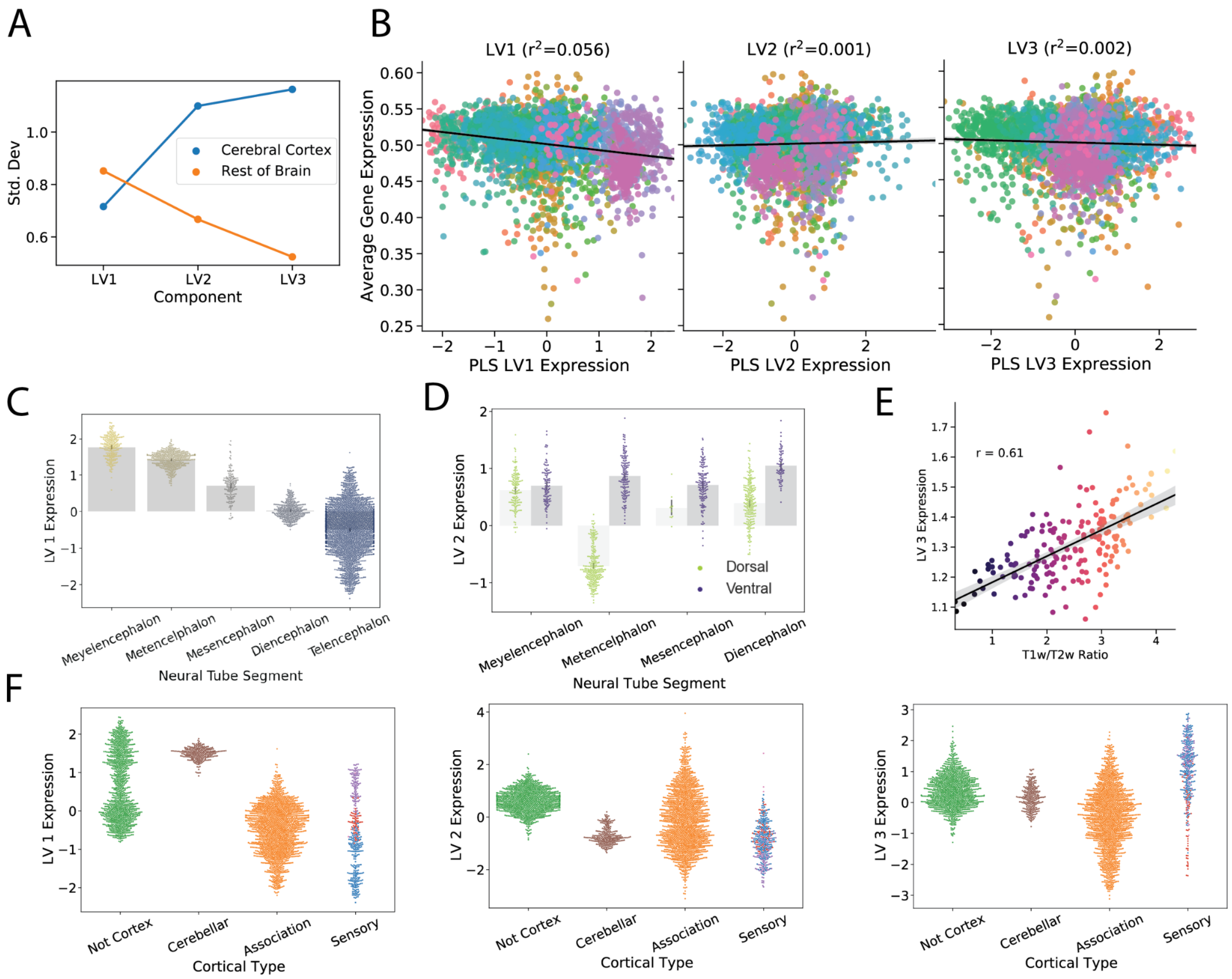

Fig S4: Spatiomolecular gradients vary systematically with fundamental developmental and cortical organizational features. **A)** Total signal variation of each component across the cerebral cortex and non-cortex, represented as the standard deviation of each component across regions. LV3 showed the greatest variation in the cerebral cortex and the least outside of it. **B)** Expression of each component correlated against total gene expression averaged across genes. Each dot represents a brain region. Dots are colored in accordance with brain division. Plots demonstrate that PLS latent variables are not simply driven by overall regional variation in gene expression. Note also that regional variation varies strongly across the X but not Y axis of each plot. **C)** Regions were divided into categories based on from which developmental compartment they originated. LV1 expression demonstrated a linear rostral-caudal gradient along developmental compartments. **D)** Within each compartment, regions were further divided based on whether they originated from dorsal or ventral aspects of the developing human brain (this information is not available for the telencephalon). Across compartments, LV2 expression differed between dorsal- and ventral-originating regions. **E)** Cortical LV3 expression correlates (r=0.78) with the T1w/T2w ratio, an MRI-derived index of myelination. **F)** All regions were categorized based on whether they were part of sensory cortex, association cortex, cerebellar cortex or non-cortex (i.e., everything else). The distribution in expression of LV1 (left), LV2 (middle) and LV3 (right) across these cortical types is visualized. Each component carries a

distinct cortical profile: LV1 expression is low in cerebral cortex but high in other types, LV2 expression is low in all cortical types but high in non-cortex, and LV3 expression is low in association cortex but high in all other cortical types. These results suggest that cortical organization is organized along the three molecular axes.

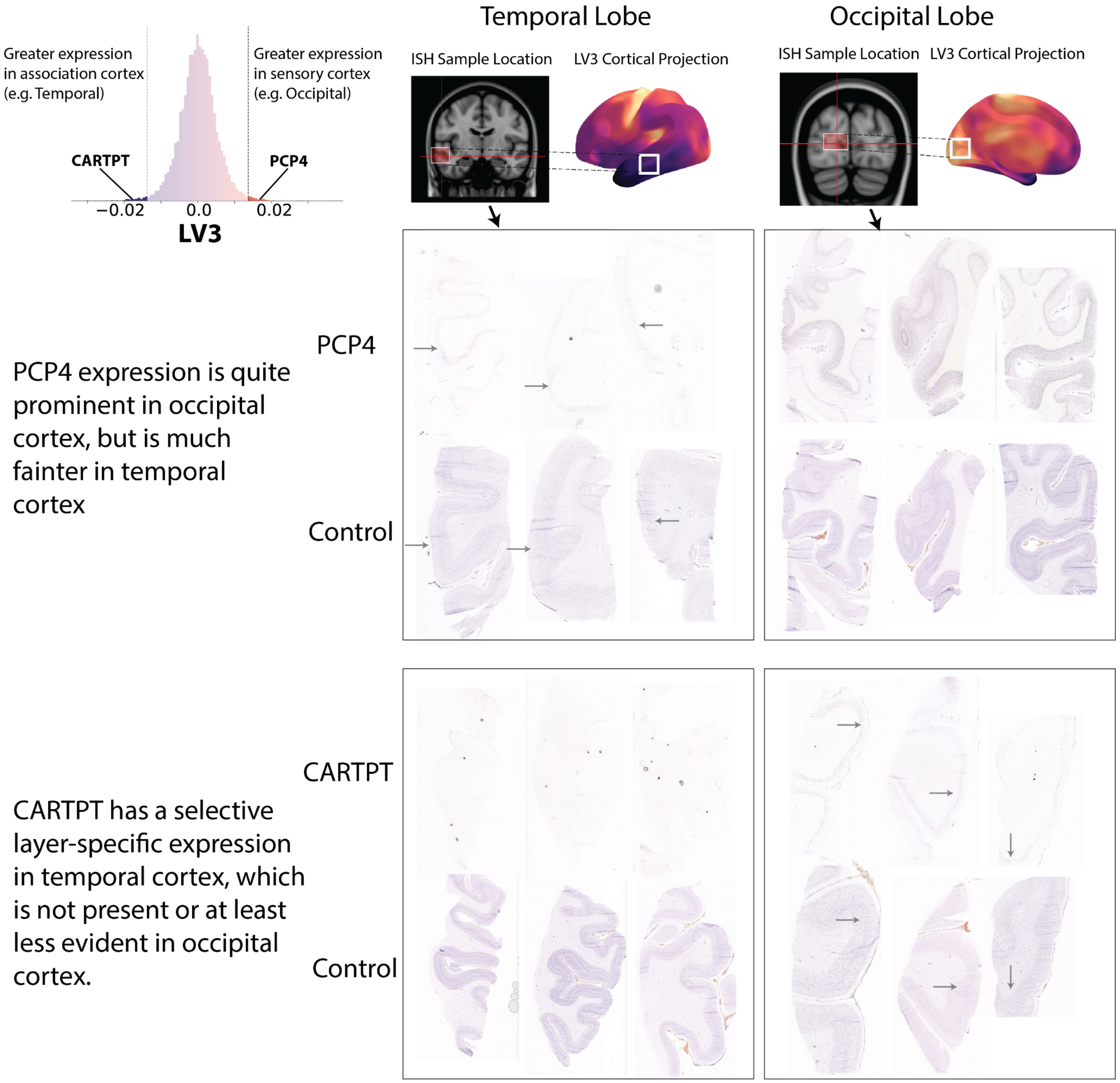

Fig S5: **Validation with *in situ* hybridization (ISH) data.** We use Allen Brain Atlas ISH data to validate the expression patterns of LV3. LV3 was chosen since it shows the most variation in the cerebral cortex, and only cortical ISH data was available. A small subset of top LV3 genes had ISH data at multiple distinct cortical areas. We used PCP4 and CARTPT as exemplar genes. For each gene, ISH-based expression of the gene is shown on top, with the nearest Nissl-stained reference image (labeled “Control”) below. Our original analysis showed PCP4 as a gene expressed in the sensory/medial (lighter) extreme of LV3, while CARTPT was expressed in the association/lateral (darker) extreme. The ISH data confirm this – PCP4 is expressed much more promiscuously in the medial occipital cortex compared to the lateral temporal lobe, while CARTPT expression was light in the lateral temporal lobe and absent from the occipital cortex.

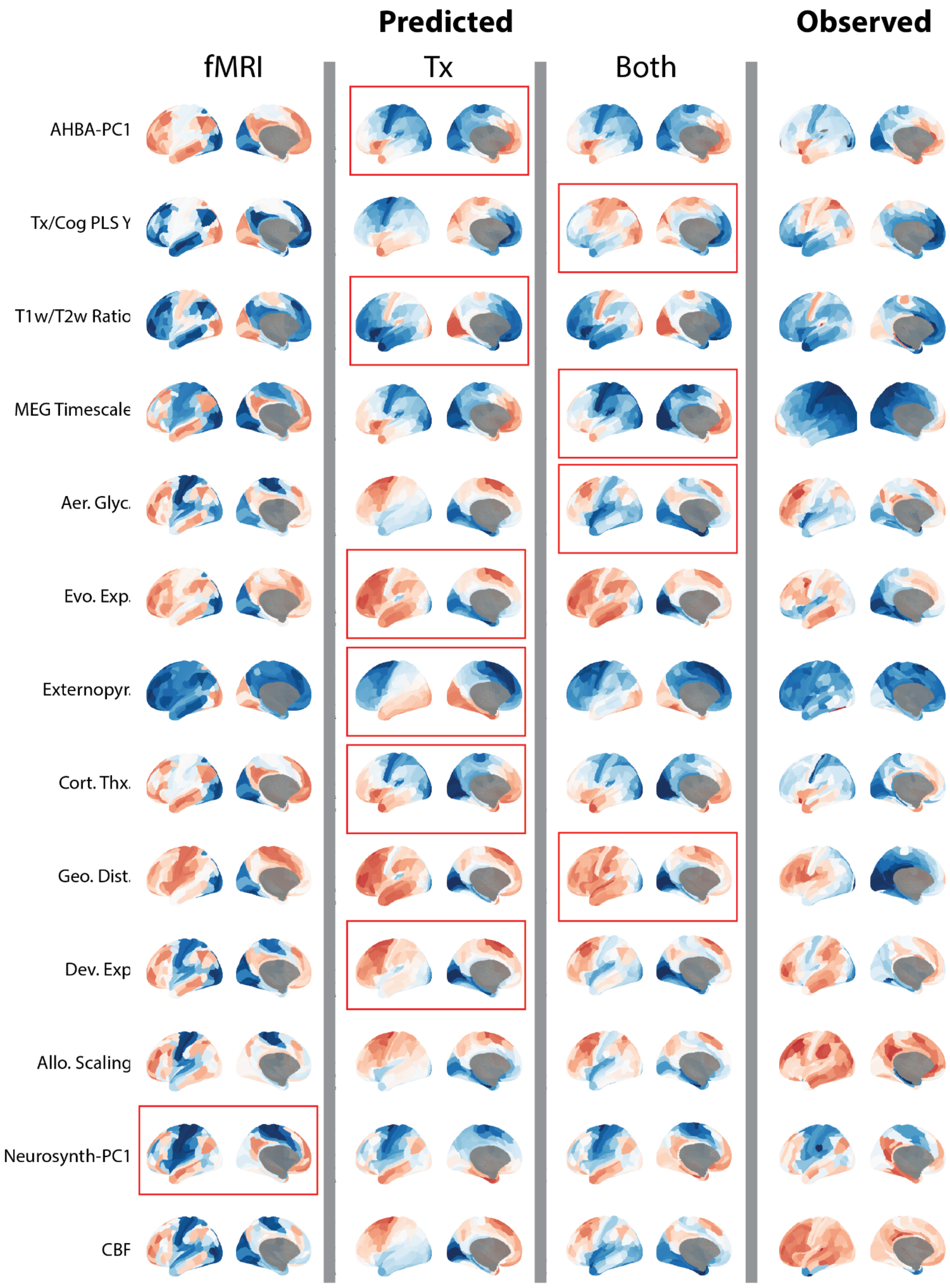

Predicted
Observed
fMRI
Tx
Both
AHBA-PC1
Tx/Cog PLS Y
T1w/T2w Ratio
MEG Timescale
Aer. Glyc.
Evo. Exp.
Externopyr.
Cort. Thx.
Geo. Dist.
Dev. Exp
Allo. Scaling
Neurosynth-PC1
CBF

Figure S6. Visualization of gradient-based model predictions of cortical properties. The observed column shows the cortical distribution of each brain feature from Fig. 2B. The predicted column shows the predicted cortical distribution of each feature based on models using the three principal fMRI gradients (left), the three latent transcriptomic gradients (middle), or both sets of gradients (right) as model predictors. Values from each image are scaled to 0 mean and unit variance. Red boxes depict the “best model”. When the “both” model performs significantly better than the fMRI and Tx models, it is selected. When the Tx model performs better than the fMRI model (but no further improvement was seen by adding fMRI features), the Tx model is selected. And vice-versa for the fMRI model. Stats and confidence intervals are shown in Main Text Figure 2B,C.

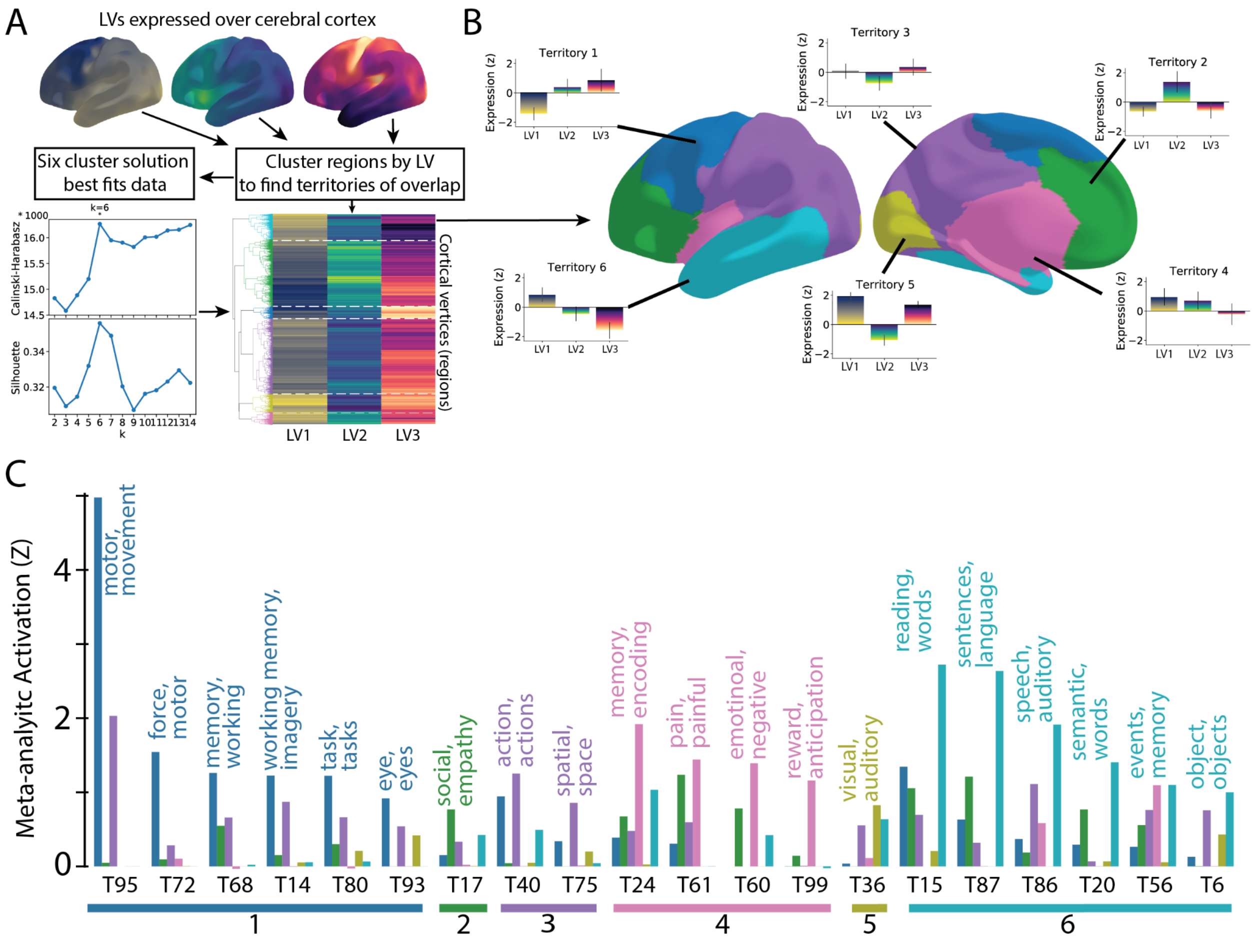

Fig S7: The interaction of functional gradients in the cerebral cortex creates functionally distinct territories. We sought to determine cortical territories defined by the interaction of whole-brain molecular gradients overlapping in the cerebral cortex. **A)** Each LV was projected to the cerebral cortex. To create an unbiased account of territories with overlapping gradients, a vertex-wide hierarchical clustering analysis was used to determine large clusters of vertices with similar LV concentrations. Cluster evaluation metrics suggested a six-cluster solution. **B)** Vertex labels of each cluster representing six molecular territories composed of different concentrations of PLS gradients. Surrounding bar plots indicate relative concentrations of each gradient within each territory. **C)** Molecular territories form functionally distinct domains: 68 cognition-associated meta-analytic topic maps were downloaded from neurosynth, representing common activation patterns across studies in association with inter-related terms. Average z-scores for each map were computed for each functional territory. Plot shows each term that fell into the top 5 highest-rank z values for one of the six molecular territories, indicating the mean z for each territory, and the top two words associated with the topic map. The x-axis shows the Topic labels, for which definitions are available at neurosynth.org and in **Dataset S8.**

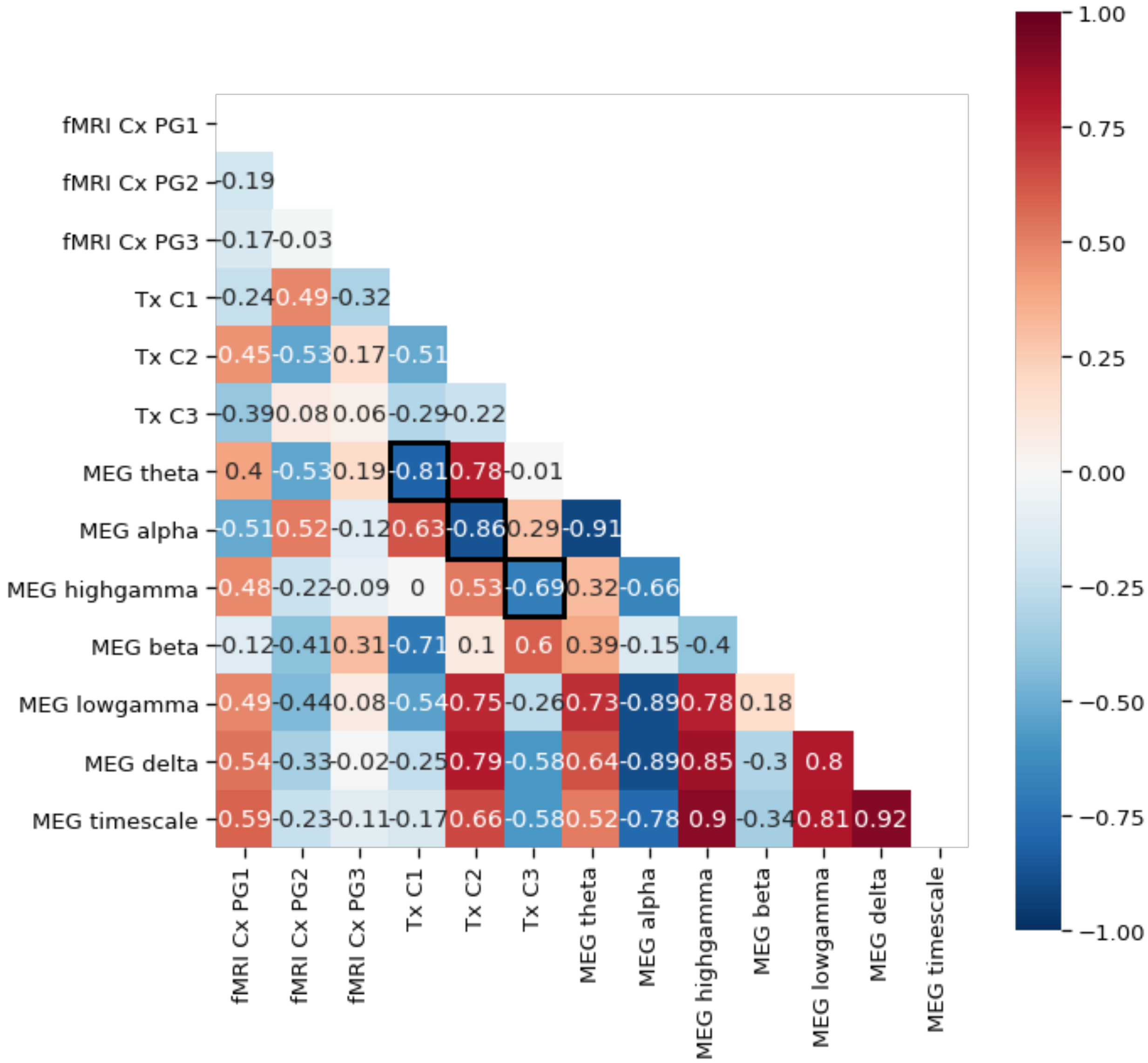

Fig S8. Spatial relationships between molecular and functional gradients of the cerebral cortex. Correlation matrix representing spatial relationship between fMRI principal gradients 1-3 (fMRI Cx PG1-3), the three molecular gradients described in this study (Tx LV1-3), and seven MEG maps (meg alpha, beta, delta, theta, low gamma, high gamma, intrinsic timescale). For each molecular gradient, the strongest relationship is highlighted with bolded box borders. These relationships are visualized in main text **Fig 2F**.

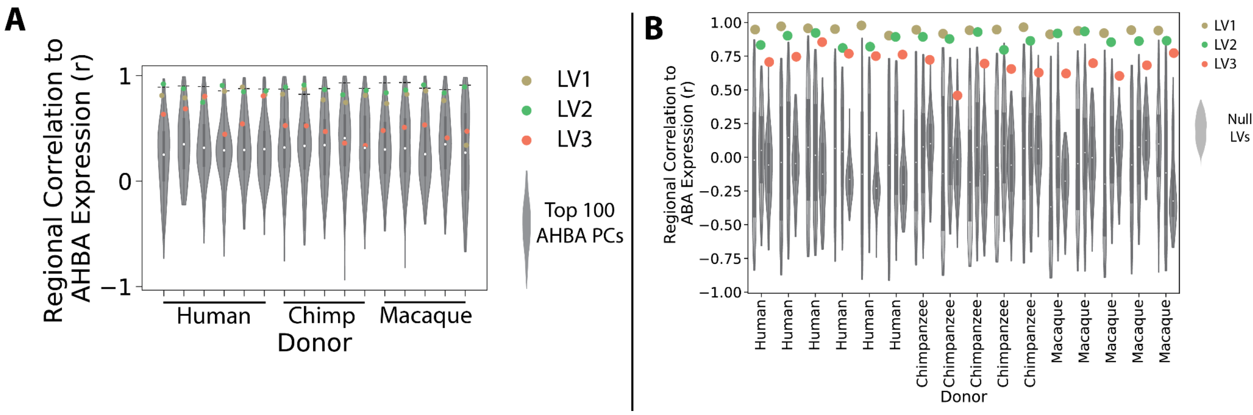

Fig S9. Individual null models for PLS latent variable replication. As described in Figure 4A, six adult human, five adult chimpanzee and five adult macaque brains were available from (http://evolution.psychencode.org; (9)) with tissue samples extracted from the same 16 brain regions. **A)** To test whether high correlations were specific to the PLS latent variables over other sets of spatially co-expressed genes, for each individual we performed correlations described in **Fig 3** for each of the first 100 principal components of AHBA gene expression data. Null distributions (gray) represent distribution of cross-dataset correlations. Horizontal lines indicate correlations that are in the top 5% of AHBA PC distribution. **B)** Component correlations (as described in **Fig 1I**) for each LV were generated for each individual. Null models were also generated for each individual. Specifically, PLS weights were shuffled 100 times for each component, and the reproducibility analysis (Fig **1I**) was conducted using each of the shuffled weights, separately for each individual. The resulting similarity (r) values were used to create a null distribution specific to each component for each individual. Null distributions are visualized, while the "true" similarity (r) values for LV1 (yellow), LV2 (green) and LV3 (orange) are represented as colored dots. These suggest that the successful replication of the PLS LVs is greater than would be expected given the structure of the data and overall similarity between datasets.

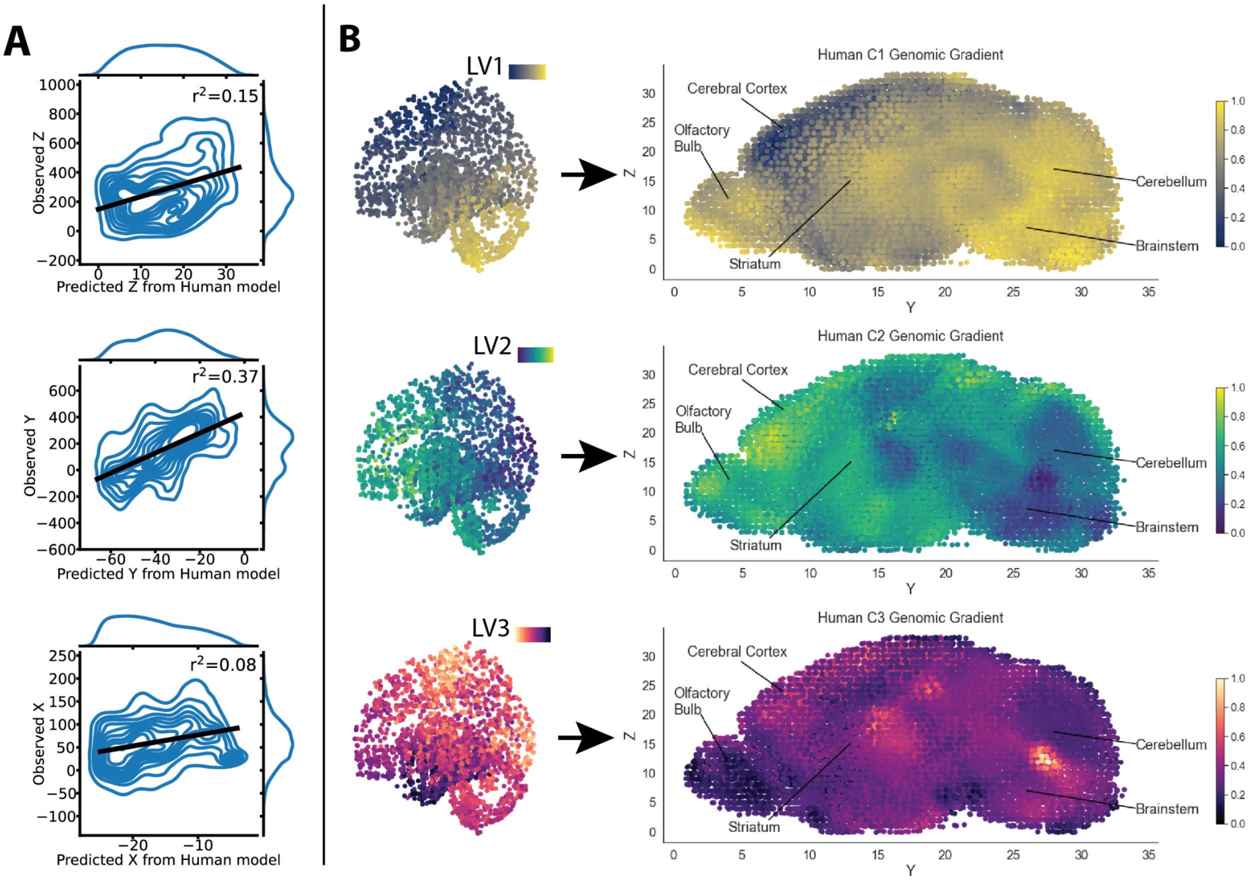

Fig S10. PLS latent variables expression in the mouse brain. A) Voxel-wise in situ hybridization data was available for the postnatal mouse brain. The PLS model trained on human data was applied to the mouse gene expression data to predict the spatial coordinates of each voxel based on gene expression. Plots compare the observed x, y and z coordinates to the coordinates predicted based on the (human) LV expression. Aerial (density) plots are used to represent voxel density. **B)** The human PLS model was applied to the mouse data, resulting in LV expression in the mouse. LV expression was projected across all voxels and displayed in a sagittal view. Expression patterns of LV1 and LV2, but not LV3, resemble human expression patterns.

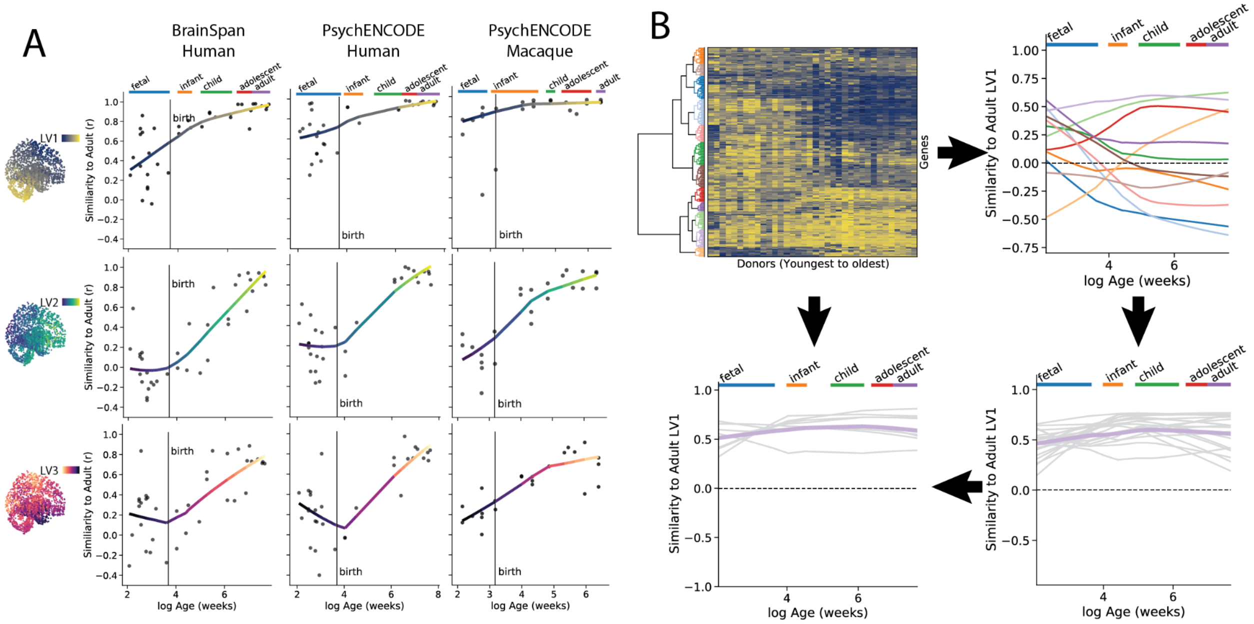

Fig S11. Individual trajectories of PLS latent variables over development. A) The same data plotted in Main Text **Fig 4A**, but separated by PLS latent variable. Here, it is possible to appreciate increased variation in component expression in early prenatal stages of human datasets, perhaps suggesting an early-prenatal peak followed by a perinatal dip. **B)** Workflow for gene-trajectory clustering, using LV1 as an example. (Top right) For each brain donor in Brainspan, the regional similarity to LV1 (**Fig 1I**) was calculated for each of the top LV1 genes. This data was then clustered to identify genes with similar developmental trajectories. (Top right) The developmental trajectory of each cluster is visualized, where the x-axis is developmental time (log of age in post-conception weeks) and the y-axis is regional expression similarity to LV1. (Bottom right) One cluster demonstrated a non-transitional trajectory with consistently high regional similarity to LV1. The individual trajectories of each contributing gene is visualized. (Bottom left) The clustering solution from Brainspan was applied to PsychENCODE data to identify genes demonstrating the same trajectory. Genes that fell into the non-transitional cluster across both datasets were considered to be reliable and are highlighted in the main text and **Fig. 4C**.

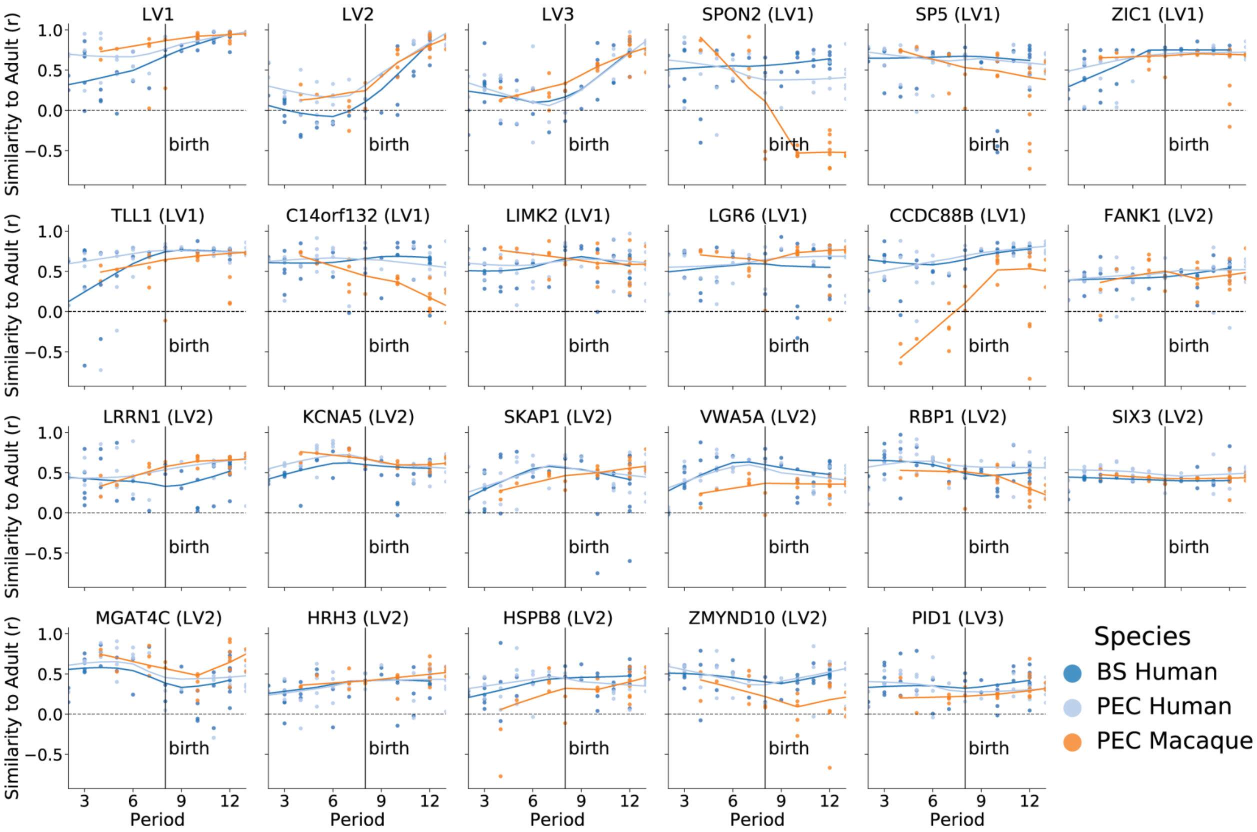

Fig S12. Comparison of candidate LV-maintenance genes across primate species. In order to establish whether genes identified as candidates for LV maintenance were consistent across datasets, it was necessary to align macaque and human developmental timelines to a shared temporal space. We used the developmental periods described in (32), and assigned the primate PsychENCODE data to these periods based on (9). For all three LVs and for each of the 21 candidate genes, we visualize regional similarity to adult (AHBA) latent variable expression across brain development. The x-axis of each plot represents developmental period and the y-axis represents similarity to adult regional LV expression. The parenthetical LV in each plot title indicates which LV is being compared. Most genes showed highly similar non-transitional developmental trajectories across species, though there were some notable exceptions (e.g. *SPON2*, *CCDC88B*).

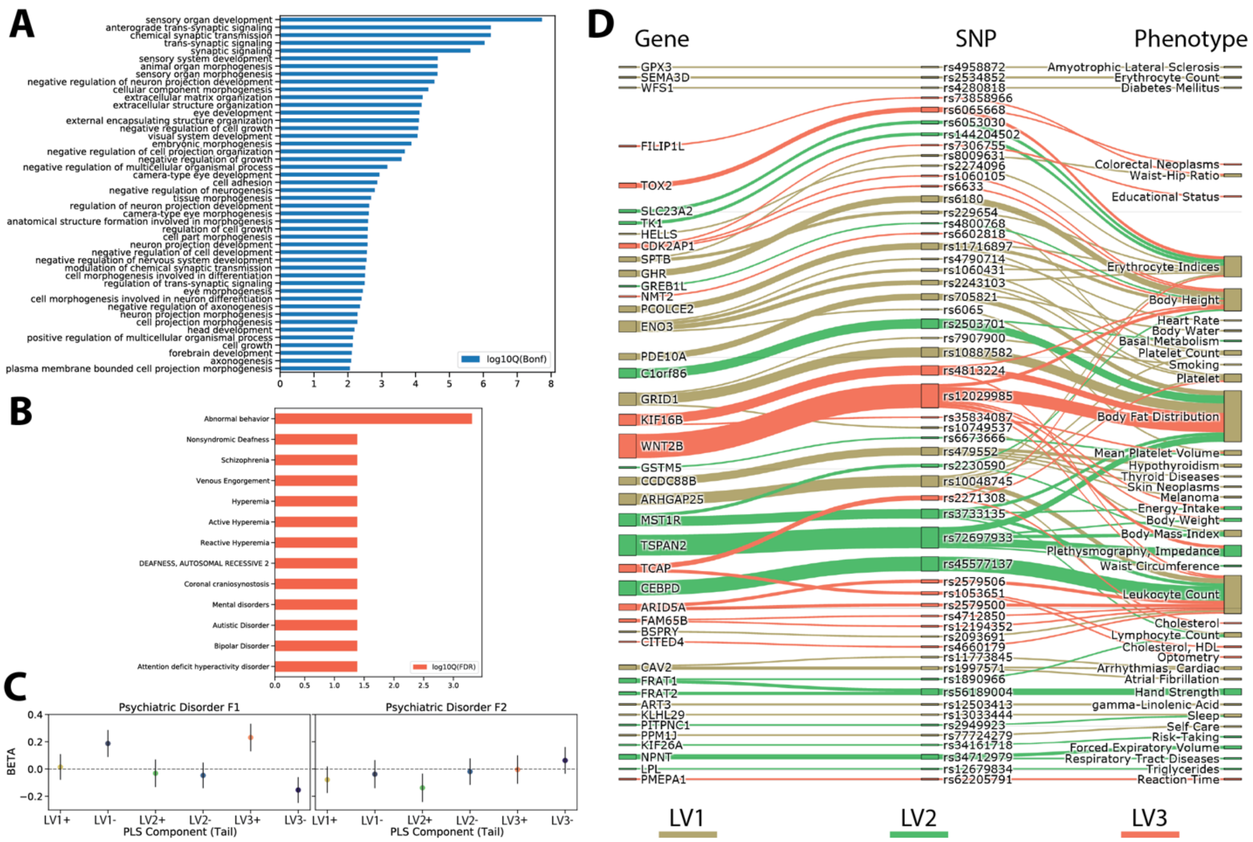

Fig S13. Annotation of gradient-associated genes. Genes composing adult LVs are associated with brain development, adult disease, and physiology. **A)** Gene set enrichment across all gradient-related genes indicated a robust enrichment for terms associated with development and morphogenesis of neural organs and cells, as well as synaptic signaling. **B)** Genes contributing to molecular gradients were also enriched for several diseases, particularly psychiatric diseases. **C)** To garner greater specificity of the effects in E), gene sets from each tail were compared to a two-dimensional GWAS of neuropsychiatric disorders. Significant associations were seen between the first dimension and both LV1 and LV3 genes. The y-axis shows the effect size (standardized beta) of the association. **D)** We identified SNPs associated with brain expression of gradient-associated genes, and found genotype significant relationships those SNPs had with various phenotypes. Sankey diagram indicates these dependent relationships, suggesting pleiotropic effects of these genes on aspects of body morphology and circulation, as well as brain-related traits.
